# Genomic Diversity, Phylogenetic Structure, and In Silico Diagnostic Primer Compatibility of Available DENV-1 Genotype V from Mexico

**DOI:** 10.3390/tropicalmed11100287

**Published:** 2026-10-12

**Authors:** Francisco Joel Cabrera-Santos, Fátima Yedith Camacho-Sánchez, Sorayda Leonela González-Trujillo, Miguel Angel Reyes-López, Erick de Jesús De Luna-Santillana

**Affiliations:** 1 Laboratory of Conservation Medicine, Centro de Biotecnología Genómica, Instituto Politécnico Nacional, Reynosa 88700, Tamaulipas, Mexico; fcabreras2100@alumno.ipn.mx (F.J.C.-S.); facamacho@uat.edu.mx (F.Y.C.-S.); sgonzalezt2400@alumno.ipn.mx (S.L.G.-T.); mreyesl@ipn.mx (M.A.R.-L.); 2 UAM Reynosa-Aztlán, Universidad Autónoma de Tamaulipas, Reynosa 88740, Tamaulipas, Mexico

**Keywords:** dengue virus, DENV-1, Mexico, genotype V, phylogenetics, purifying selection, molecular diagnostics

## Abstract

**Background:** Dengue virus serotype 1 (DENV-1) is prevalent across the Americas, yet its genomic diversity in Mexico is poorly characterized. **Methods:** We analyzed 119 public Mexican DENV-1 genomes (polyprotein CDS, 10,179 nt) alongside 111 from 19 other American countries and territories. The Mexican panel was temporally and geographically uneven (91% from 2006 to 2009), representing available sequences, not nationwide diversity. We assessed diversity, phylogeny, dating, selection, recombination, and compatibility with three primer sets. **Results:** The Mexican genotype V sequences formed 115 haplotypes (Hd = 0.9994; π = 0.00831), resolving into clades A (n = 6) and B (n = 112) plus one independent introduction in Jalisco. These sequences were not monophyletic: both clades grouped with Nicaraguan and United States viruses. Their common ancestor dated to 1977.5 and clade divergence to 1984.2. Selection appeared to be predominantly purifying (ω = 0.037); no site showed concordant evidence of diversifying selection, and no recombination was detected. In silico, 84% of Mexican sequences were compatible with all three CDC real-time oligonucleotides; none matched every oligonucleotide in either conventional set. **Conclusions:** This historical panel shows substantial haplotype diversity and multiple introductions. The compatibility results are computational and were not validated experimentally. We recommend periodic in silico primer revalidation.

## 1. Introduction

Dengue virus (DENV) comprises four antigenically distinct serotypes (DENV-1 to DENV-4) and remains a major global public health concern, with an estimated 390 million infections annually [1]. In the Americas, DENV-1 is among the prevalent serotypes; however, comprehensive genomic characterization of this serotype in Mexico remains limited. Genetic diversity within DENV-1, particularly genotype V, has been linked to epidemic potential and disease severity [2,3]. According to the Pan American Health Organization (PAHO) epidemiological update of 29 March 2024, approximately 4.57 million dengue cases were reported in the Americas in 2023 [4]. This escalating burden underscores the need for enhanced genomic surveillance.

Recent studies indicate that DENV-1 genotype V has been associated with large outbreaks in South America [5,6] and is the predominant genotype circulating in Mexico [2,3]. However, the fine-scale haplotype structure and phylogeographic patterns within Mexico remain poorly understood. Understanding these patterns is essential for (i) tracking viral introduction and dispersal routes [7,8]; (ii) identifying lineages associated with severe disease [9]; (iii) informing vaccine development and deployment strategies [10]; and (iv) enhancing outbreak prediction and response [11].

Genomic surveillance has proven invaluable in other Latin American countries. For example, in Colombia, phylogeographic analyses of 11,443 genomes revealed repeated introductions and exportations of DENV-2 and DENV-3 lineages, highlighting the region’s role in viral dispersal [12]. Similarly, in Uruguay, genomics-based detection enabled the prompt identification of multiple co-circulating DENV-1 genotypes, facilitating timely public health responses [13].

In Mexico, however, genomic surveillance has largely been limited to urban centers, leaving remote and Indigenous regions, where dengue is increasingly reported, without molecular characterization [14,15]. The Sierra Tarahumara, for instance, has documented the presence of *Aedes aegypti* but lacks genomic data from circulating viruses [15]. This gap limits our understanding of viral diversity and dispersal in areas that may serve as reservoirs or sources of novel variants. The uneven geographical and temporal distribution of the currently available Mexican sequences, dominated by a single state and a single outbreak period, further constrains the inferences that can be drawn from public data and must be stated explicitly when such data; this limitation must be stated explicitly when such data are used for national-scale characterization. Bioinformatics approaches, including haplotype reconstruction and selection pressure analysis, can bridge this gap by leveraging existing genomic data to infer patterns that guide targeted sampling.

Therefore, this study aimed to characterize the genomic diversity, haplotype structure, and phylogenetic history of the available Mexican DENV-1 sequences using all publicly available complete coding sequences. We also dated the divergence of the recovered lineages and evaluated in silico whether the sampled sequences are compatible with three diagnostic oligonucleotide sets. Because the analysis relies exclusively on public sequences, the results describe the diversity captured by existing repositories rather than a designed national sampling; this limitation is stated throughout and does not reduce the utility of the approach. Where local sequencing capacity is limited, public data and open-source bioinformatics tools can still support meaningful characterization and identify priority targets for future surveillance. Finally, this study provides a reproducible framework that can be applied to other DENV-endemic settings where local sequencing capacity is limited but public data are available.

## 2. Materials and Methods

### 2.1. Dataset

The expanded phylogenetic dataset comprised 245 complete coding sequences: 119 Mexican DENV-1 sequences, 111 DENV-1 sequences from 19 other American countries and territories, 14 DENV-1 genotype reference sequences, and 1 DENV-2 outgroup sequence. Four additional partial NS5 sequences from a previously published Mexican study were used only for clade assignment and were not included in the phylogenetic alignments (Table S8).

All DENV-1 genome sequences associated with Mexico were retrieved from GenBank (NCBI) with the rentrez package (v1.2.4) [16] on 18 August 2026 using the query txid11053 [Organism:exp] AND Mexico [All Fields] AND 9000:11,000[SLEN], which returned 134 records. Records were then filtered using the annotated/country qualifier instead of free-text matching, which excluded one non-Mexican record and yielded 133 Mexican genomes. Polyprotein coding sequences (CDS) were obtained directly from the GenBank annotation. Sequences were retained if the annotated CDS was exactly 10,179 nt long, the canonical DENV-1 polyprotein length (n = 127), and if ambiguous positions represented no more than 1% of the CDS (n = 119). Six sequences were excluded for length and eight for ambiguity content, which ranged from 1.6% to 16.2%. Accession numbers, collection years, and states for the 119 retained sequences are provided in Supplementary Table S1.

The 14 DENV-1 genotype reference strains and the DENV-2 outgroup (NC_001474.2) are listed in Table S2. The 111 American context sequences originated from 19 countries and territories and were retrieved and filtered using the same criteria. Geographic origin was obtained from the GenBank/geo_loc_name qualifier or, when unavailable, the /country qualifier. Records with known collection years were subsampled to at most three per country or territory and year. Records without an obtainable collection year were treated as a separate group within each country or territory, with at most five retained before applying the overall limit of 25 records per country or territory. The final contextual dataset contained 15 records without an obtainable collection year. Accession numbers, geographic origins, and collection years are listed in Table S5, and regional composition is summarized in Table S1. The four partial NS5 sequences from González-Durán et al. [3] are listed in Table S8.

### 2.2. Alignment and Sequence Curation

Because all retained CDS were uniformly 10,179 nt long and DENV-1 polyproteins lack intra-serotype insertions or deletions, the sequences were stacked directly without gap-inserting alignment. This preserves the reading frame by construction and avoids the frame displacement that arises when gapped columns are removed from a nucleotide alignment. Three independent checks supported this approach. All 119 sequences contained start and stop codons and encoded a 3392-residue polyprotein without internal stops. Alignment with MUSCLE ver. 3.54.0 [17] yielded a gap-free alignment identical to the directly stacked matrix. Gene boundaries were assigned from polyprotein coordinates and independently validated by locating the conserved NS5 GDD motif (residue 3154) and the NS3 DEAH helicase motif (residue 1760) within their expected genes.

### 2.3. Phylogenetic Reconstruction

The 119 Mexican coding sequences were combined with 14 DENV-1 reference strains spanning all five genotypes and with the DENV-2 reference genome NC_001474.2 as an outgroup. To place the Mexican sequences within regional diversity, an expanded alignment additionally included the 111 DENV-1 contextual sequences retrieved, filtered, and subsampled as described in Section 2.1. The resulting set covers four Mesoamerican origins (28 sequences), six Caribbean origins (31), eight South American origins (46), and the United States (6); its full composition is given in Table S1. Because DENV-1 and DENV-2 polyproteins differ by three single-codon indels, these were positioned by aligning the translated DENV-1 consensus against the translated outgroup with MUSCLE and propagating the resulting gaps to the corresponding codons. Maximum-likelihood inference was performed in IQ-TREE v3.1.0 [18], with the best-fitting substitution model selected by ModelFinder ver. R2026b [19] under the Bayesian information criterion (TIM2+F+I+R2 for the Mexican alignment and TIM2+F+I+R3 for the expanded alignment). Branch support was assessed with 1000 ultrafast bootstrap replicates [20], including NNI refinement, and 1000 replicates of the SH-like approximate likelihood ratio test [21]; bootstrap convergence was verified in both analyses. Branches were considered supported when ultrafast bootstrap values were at least 95 and SH-aLRT values were at least 80. Genotype assignment was additionally verified using an approach independent of tree topology. For every Mexican sequence, the mean uncorrected *p*-distance to each genotype reference set was computed, and the sequence was assigned to the nearest set. To place partial sequences that could not enter the main alignment, each of the 121 positions distinguishing the two Mexican clades was represented by the 21-nucleotide clade consensus centered on it, and a partial sequence was assigned to the clade whose context sequences it contained; the procedure was validated on sequences of known clade membership, which it classified without error. Clades were delimited as the maximal nodes subtending at least five sequences, all of them Mexican, that met the support criterion. Temporal signal was assessed before dating by root-to-tip regression, computed in R from the rooted maximum-likelihood tree with the ape package, and was confirmed by a date-randomization test in which collection years were permuted at random across tips and dating was repeated twenty times under the same model and topology. The Mexican tree was dated with LSD2 v2.4.4 [22] as implemented in IQ-TREE using collection year for calibration and 100 resampling replicates for confidence intervals.

### 2.4. Haplotype Network Analysis

Haplotypes were identified, and a minimum spanning haplotype network was constructed with the pegas (v1.4) [23] and ape (v5.8.1) [24] packages in R (v4.5.2) [25]. Node area was scaled to haplotype frequency, and node color encoded either the state of origin or the collection year. To accommodate the wide range of mutational distances across the network, the topology was rendered using a force-directed algorithm [26], compressing link lengths for visual clarity rather than scaling them to reflect genetic distance. This representation prioritizes topological relationships among haplotypes; quantitative distance information is not encoded in branch length, and this constraint is explicitly noted in the legend. Haplotype identity was confirmed by direct sequence comparison, since ambiguity codes can cause distinct sequences to be merged. Of four candidate shared haplotypes, three were identical at all positions, and one pair differed at a single position at which one sequence carried an ambiguity; this pair was therefore treated as unresolved. Genetic diversity indices were computed in R ver. 4.6.2.

### 2.5. Neutrality Tests and Selection Pressure Analysis

Neutrality statistics (Tajima’s D [27], Fu and Li’s D* and F* [28], and Fu’s Fs [29]) were computed in R following the conventions of DnaSP v6.12.03 [30], which bases these statistics on the total number of mutations rather than on the number of segregating sites; the implementation was validated by reproducing the published DnaSP output for the original panel exactly. Rates of synonymous (dS) and non-synonymous (dN) substitution were estimated for each mature protein and for the complete polyprotein by the Nei-Gojobori counting method [31] with Jukes-Cantor correction [32], averaged over all 7021 pairwise comparisons; codons containing an ambiguous base in any sequence were excluded (41 of 3392 codons). Because pairwise comparisons are not statistically independent, no significance test is reported for the pairwise ratios. Codon-model analyses were performed in HyPhy v2.5.101 [33] using the maximum-likelihood tree described above rather than a tree inferred de novo. Site-level selection was assessed with three methods that differ in their inferential framework: FEL [34] and MEME [35] (likelihood-based methods that test for pervasive and episodic selection, respectively) and FUBAR [36] (Bayesian). Sites were considered supported only when identified by at least two of the three methods, using *p* < 0.05 for FEL and MEME and a posterior probability above 0.9 for FUBAR. Gene-wide episodic selection was tested with BUSTED [37]. Selection specific to clade A was tested by marking its branches as foreground and applying aBSREL [38], which tests individual branches for episodic diversifying selection, and RELAX [39], which tests whether selection intensity differs between foreground and background branches. Recombination was screened for using GARD [40] on the codon alignment, as intra-serotype recombinants have been reported in dengue virus [41] and undetected recombination can bias both phylogenetic and selection inference.

### 2.6. In Silico Primer Compatibility Analysis

Complementarity between the Mexican DENV-1 sequences and three diagnostic oligonucleotide sets was evaluated in silico. The first was the conventional serotyping system based on the NS3 primers of Seah et al. [42], for which the DV1 and DSP1 sequences were taken from Domínguez de la Cruz et al. [43] (DV1, forward, 5′-GGRACKTCAGGWTCTCC-3′; DSP1, reverse, 5′-AGTTTCTTTTCCTAAACACCTCG-3′). The second was the DENV-1 component of the CDC DENV-1–4 Real-Time RT-PCR Assay [44] (D1-F, forward, 5′-CAAAAGGAAGTCGYGCAATA-3′; D1-R, reverse, 5′-CTGAGTGAATTCTCTCTGCTRAAC-3′; D1-Probe, 5′-CATGTGGYTGGGAGCRCGC-3′), using the modified versions with degenerate positions reported in that study rather than the earlier sequences presented there for comparison. The 2025 WHO interim guidance on laboratory testing for dengue recommends nucleic acid amplification testing for acute-phase diagnosis and the use of validated primers and probes, but does not prescribe a single universal oligonucleotide set; it states that dengue in vitro diagnostics were not then part of the WHO prequalification portfolio and that an Expert Review Panel for Diagnostics had been established to evaluate and recommend products [45]. Commercial diagnostic products evaluated through that panel were outside the scope of this study. Earlier WHO guidance instead describes a nested RT-PCR targeting the C/prM region and cites the assay of Lanciotti et al. [46]. The third set was therefore the DENV-1 component of that historical assay, comprising the consensus primers D1 and D2 and the type-specific primer TS1 [47] (D1, 5′-TCAATATGCTGAAACGCGCGAGAAACCG-3′; D2, 5′-TTGCACCAACAGTCAATGTCTTCAGGTTC-3′; TS1, 5′-CGTCTCAGTGATCCGGGGG-3′).

Binding sites were located by sliding each oligonucleotide and its reverse complement across the alignment and selecting the window with the fewest discordances; the runner-up window was recorded across both orientations to confirm that each assignment was unambiguous. Each position was then classified by comparing the set of bases permitted by the oligonucleotide with the set possible at the target: a target set contained within the oligonucleotide set was counted as compatible, a disjoint pair as incompatible, and partial overlap as indeterminate. Compatibility is reported under this set-based criterion and, separately, restricted to targets with fully resolved bases. For each sequence, we recorded the number of discordant positions, their position relative to the oligonucleotide 3′ terminus, the number of contiguous compatible bases at that terminus, and the observed codon. For hydrolysis probes, the count of contiguous 3′ bases is not reported, because the 3′ end carries the quencher and is not extended. Product sizes predicted from the primer positions were compared with the published sizes as a check, and the implementation was verified against the deposited per-sequence table for the DV1/DSP1 system. Clade membership was inferred phylogenetically, as described in Section 2.3. Variant distributions across clades are therefore reported descriptively and are not interpreted as independent confirmatory evidence of association, since sequences within a clade share ancestry and are not independent replicates. No experimental validation was performed; all primer compatibility results are computational predictions, and this limitation is stated explicitly in the discussions section.

## 3. Results

### 3.1. Dataset Description and Sequence Characteristics

A total of 133 Mexican DENV-1 genomes were retrieved, of which 119 met the length and ambiguity criteria and were retained for analysis. Sequences were collected from 2006 to 2019. Sixty-six sequences were from Yucatan, two from Jalisco, and one from Quintana Roo, whereas 50 lacked state-level annotation. Ninety-one percent of the panel was collected between 2006 and 2009, and only 11 sequences were collected from 2011 onwards. The analyzed coding alignment comprised 10,179 nt; after excluding 96 positions with an ambiguous base in any sequence, the remaining 10,083 nt contained 1225 segregating sites. These 96 positions fell within 41 codons, which were excluded from the codon-based analyses reported in Section 3.5.

### 3.2. Genetic Diversity and Haplotype Structure

Among the 119 Mexican DENV-1 sequences, 115 haplotypes were identified (h = 115), with haplotype diversity Hd = 0.9994 and nucleotide diversity π = 0.00831 (Table 1). Nucleotide diversity was approximately an order of magnitude lower than estimates obtained from globally sampled multi-genotype DENV-1 panels, and the difference highlights that diversity indices must be reported for an explicitly defined population. Haplotype diversity approaching unity is an expected property of whole-genome viral datasets and typically scales with alignment length. Therefore, it should not be interpreted as evidence of exceptional diversity, nor should it be compared across studies that target different genomic regions. Three haplotypes were each shared by two distinct Mexican isolates, a pattern consistent with local circulation but insufficient by itself to establish sustained transmission; a fourth candidate pair differed at a single ambiguous position and was therefore treated as unresolved. Because an ambiguous base is compatible with either alternative, this fourth pair was merged into the count of 115 haplotypes; under strict identity at every position, the panel contained 116 haplotypes.

### 3.3. Phylogenetic Structure

**Figure 1:**
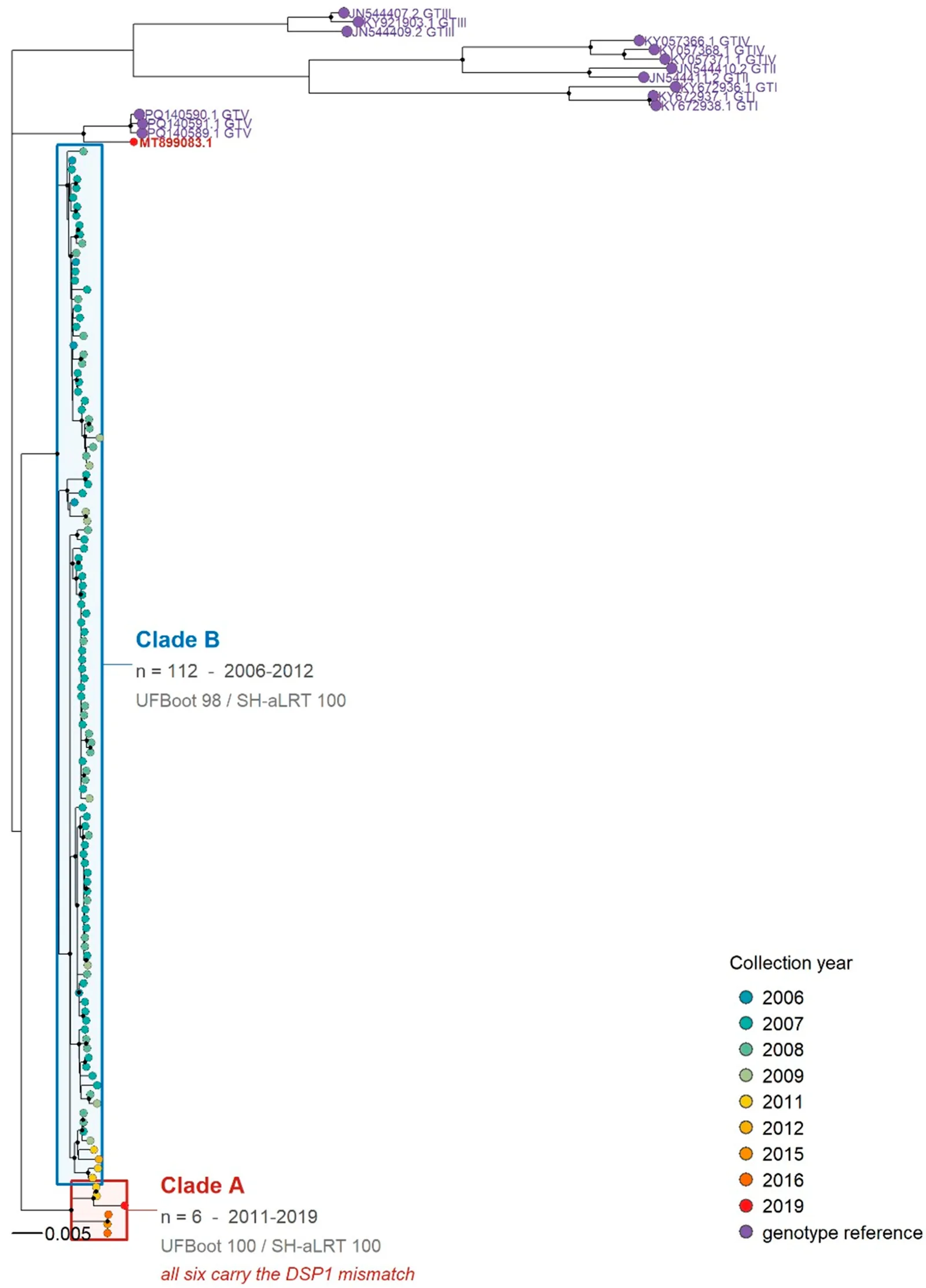
Maximum-likelihood phylogenetic tree of 119 Mexican DENV-1 sequences and 14 genotype reference strains, based on the polyprotein coding sequence (10,179 nt) under the TIM2+F+I+R2 model. The tree was rooted using the DENV-2 outgroup NC_001474.2, which is omitted because its branch length compresses the ingroup. Nodes with ultrafast bootstrap support of at least 95 and SH-aLRT of at least 80 are marked. Tips are colored by collection year; the 14 genotype reference strains are shown in purple. Clades A and B are indicated; MT899083.1 (Jalisco, 2019) groups with the genotype V reference strains rather than with the Mexican clade.

All 119 Mexican sequences fell within the portion of the tree that contained the genotype V reference strains, to the exclusion of genotypes I to IV, confirming their assignment to genotype V (Figure 1). The node uniting the Mexican sequences with the genotype V reference strains was only weakly supported (UFBoot 57); therefore, the precise relationship between the Mexican lineage and the reference strains was not resolved by these data. 118 of the 119 sequences formed a single clade (UFBoot 93, SH-aLRT 91.2) containing two maximal, well-supported subclades. The support for this basal node fell just below the 95% ultrafast bootstrap threshold used here, rendering it provisional; both subclades within it, which contained the lineages analyzed below, exceeded this threshold. Clade B comprised 112 sequences collected between 2006 and 2012 (UFBoot 98, SH-aLRT 100), and clade A comprised six sequences collected between 2011 and 2019 (UFBoot 100, SH-aLRT 100). The single exception, MT899083.1 from Jalisco in 2019, grouped with the genotype V reference strains rather than with the Mexican clade (UFBoot 97, SH-aLRT 100), consistent with an independent introduction. Overall, 54 of 131 internal nodes satisfied the combined support criterion. The presence of a single Mexican clade encompassing nearly all sequences is more consistent with a limited number of introductions followed by local expansion than with repeated independent introductions. The molecular clock analysis estimated a substitution rate of 4.61 × 10^−4^ substitutions per site per year (95% CI 3.27 × 10^−4^ to 5.53 × 10^−4^). The root-to-tip regression used to verify temporal signal yielded a somewhat steeper slope (5.89 × 10^−4^, Figure S1); the two were reported separately because the regression was a descriptive check that treated tips as independent observations and therefore tended to overestimate the rate, whereas the value used for dating was the model-based estimate. The same analysis placed the most recent common ancestor of all Mexican sequences at 1977.5 (95% CI 1965.5–1984.5), coinciding with the documented introduction of DENV-1 into the Americas. Clades A and B diverged in 1984.2 (95% CI 1974.2–1989.6), whereas their respective common ancestors dated to 2001.8 and 1998.3. The estimated divergence of clade A from clade B therefore predates the estimated common ancestor of its sampled members by approximately 17 years. The earliest clade A genome in the analyzed panel was collected in 2011; these dates do not establish when the lineage entered Mexico or whether it circulated continuously there during the intervening period.

Two independent checks supported these estimates. In a date-randomization test, in which collection years were permuted across tips and dating was repeated twenty times on the same topology and model, no replicate yielded no replicate a rate that approached the observed value: the highest randomized rate was 2.73 × 10^−5^ substitutions per site per year, roughly seventeen-fold below the estimate obtained with the true dates, and in fourteen of the twenty replicates the rate collapsed to the lower bound imposed by the optimizer, which indicated the absence of any temporal structure once the dates were shuffled. Separately, because the node uniting the Mexican sequences with the genotype V reference strains was weakly supported, genotype assignment was repeated using a criterion independent of topology: the mean uncorrected *p*-distance from each Mexican sequence to each genotype reference set. All 119 sequences were closest to the genotype V references (mean distance 0.031), whereas the next nearest genotype was more distant (genotype III, 0.057), with a minimum margin of 0.024 across the panel; thus, assignment to genotype V did not depend on the weakly supported node.

**Figure 2:**
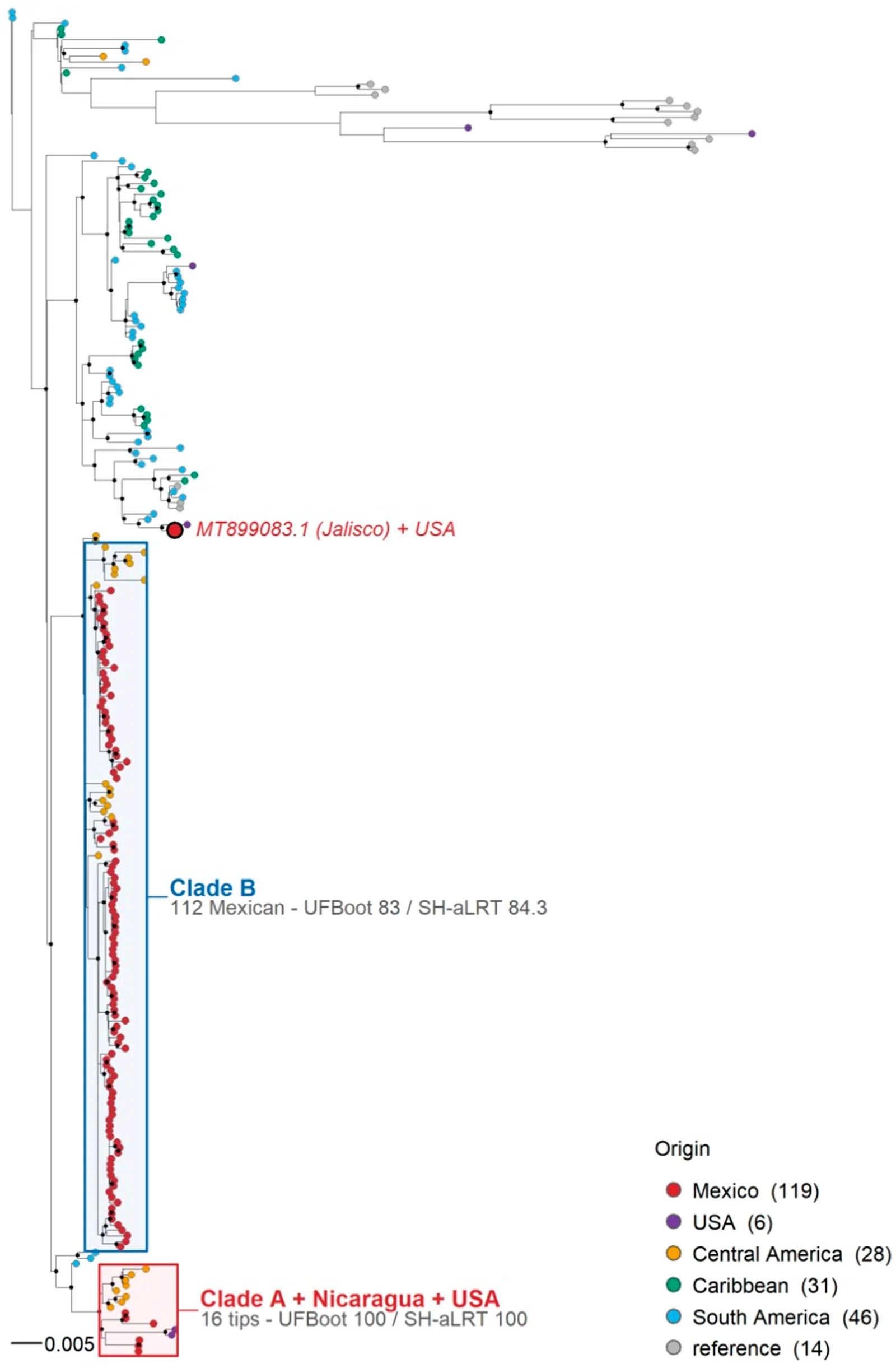
Maximum-likelihood phylogenetic tree of 119 Mexican DENV-1 sequences analyzed together with 111 sequences from 19 other American countries and territories and 14 genotype reference strains (245 taxa, TIM2+F+I+R3). Tips are colored by geographic origin. The clade uniting clade A with sequences from Nicaragua and the United States is highlighted, as is clade B; MT899083.1 is marked. The tree was rooted using the DENV-2 outgroup, which is omitted from the figure.

The inclusion of the regional context provided further insight into these relationships. In the expanded analysis of 245 sequences, the Mexican sequences were not monophyletic: their most recent common ancestor subtended 217 tips, interleaved with 95 contextual sequences from 15 other American countries and territories and three genotype V reference strains, and that node was only weakly supported (UFBoot 79). Clade A was no longer recovered in isolation. Its sister group comprised eight sequences from Nicaragua (UFBoot 93), and the node uniting clade A with those sequences and with two sequences from the United States received maximal support (UFBoot 100, SH-aLRT 100). Two of the United States sequences fell immediately adjacent to the isolate designated Ciudad Juarez. Clade B was likewise sister to two Nicaraguan sequences (UFBoot 100). Isolate MT899083.1, the Jalisco isolate that fell outside the Mexican clade, was recovered as the sister sequence to a United States sequence with maximal support (UFBoot 100, SH-aLRT 100). Within the sampled dataset, Mexican DENV-1 sequences form part of a wider Mesoamerican and North American pool rather than a distinct national lineage. However, this conclusion should be interpreted in light of the composition of the contextual sequence dataset, which is detailed in full in Table S1. Mesoamerica, the Caribbean, and South America were represented by 28 sequences from four origins, 31 from six, and 46 from eight, respectively, but sampling within the regions was uneven: 25 of the included sequences were from Nicaragua, reaching the per-country subsampling cap and exceeding the number from any other origin, whereas six were from the United States. Therefore, while the grouping of Mexican, Nicaraguan, and United States sequences received strong support, the data did not support identifying any single country as the immediate source. These relationships are shown in Figure 2.

### 3.4. Haplotype Network and Regional Connectivity

**Figure 3:**
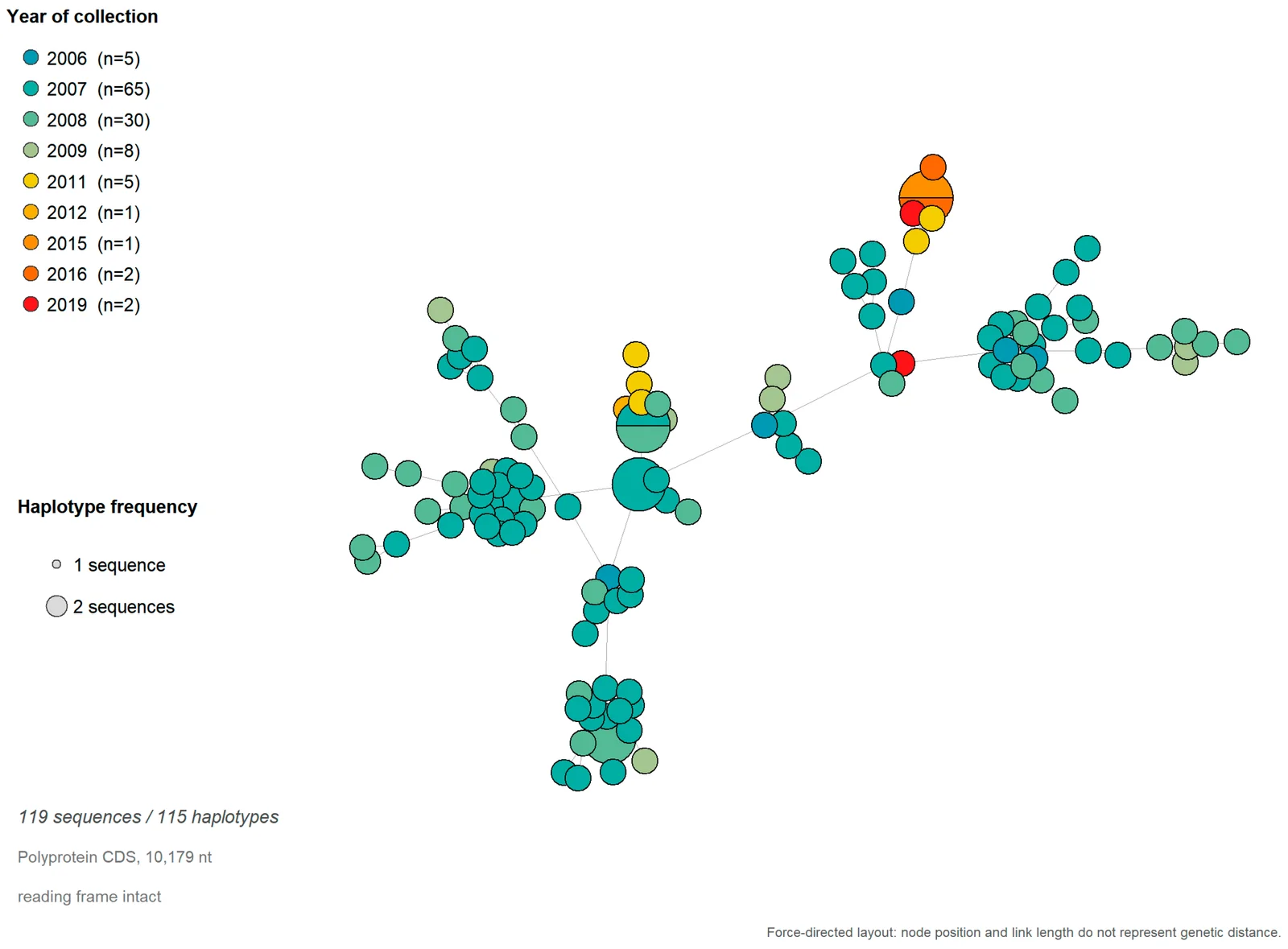
Minimum spanning haplotype network of 119 Mexican DENV-1 sequences. Node area is proportional to haplotype frequency, and node color indicates collection year. A force-directed layout was used, and link lengths were compressed for legibility; therefore, link length does encode genetic distance.

The haplotype network identified 115 haplotypes among the 119 sequences (Figure 3). Three haplotypes were each shared by two distinct Mexican isolates, whereas a fourth candidate pair, comprising Ciudad Juarez (MZ343259.1) and isolate PX282464.1, differed at a single position at which one sequence contained an ambiguity; therefore, their relationship could not be confirmed, and the pair was treated as unresolved. When the network was colored by collection year, the sequences collected from 2011 onward formed a group distinct from samples collected between 2006 and 2009, which comprised most of the panels. This temporal structure corresponded to the two clades identified in the phylogenetic analysis (Figure 3).

### 3.5. Neutrality Tests and Selection Pressure Analysis

All four neutrality statistics were negative and, unlike those reported for globally sampled panels, significantly so (Table 2). Tajima’s D was −2.18 (*p* = 0.009), Fu and Li’s D* was −4.38 (*p* < 0.05), and F* was −4.03 (*p* < 0.05). Fu’s Fs was −45.3 (*p* < 0.001). Fu’s Fs is reported for completeness and was not interpreted as independent evidence of demographic expansion. A significantly negative Tajima’s D is consistent with recent population expansion, with purifying selection, or with both; given the genome-wide substitution-rate ratio reported below, purifying selection cannot be excluded as a sufficient explanation.

Synonymous substitution rates exceeded non-synonymous rates in every mature protein and across the complete polyprotein (Table 3). The genome-wide ratio was ω = 0.037, and no gene approached neutrality: the highest ratios were observed in NS2A (ω = 0.127) and the capsid (ω = 0.134), and the lowest in NS4B (ω = 0.005). The envelope gene yielded ω = 0.023, and NS1 yielded ω = 0.017, corresponding to synonymous rates 44-fold and 59-fold higher than non-synonymous rates, so the two genes most often proposed as targets of immune-driven diversification were among the most constrained in this dataset.

Codon-model analyses confirmed this pattern and found no support for diversifying selection at any site. Across 2365 codons from the envelope, NS1, NS3 and NS5 genes, five sites were flagged by a single method (NS1 codons 60 and 146 and NS5 codon 28 by FUBAR; NS3 codon 1 and NS5 codon 128 by MEME), and none was identified by two or more methods. Because the three methods differ in their inferential framework and each has a different false-positive rate, concordance among them was the relevant criterion, but no site met it. In the envelope gene, no site was flagged by any method. Purifying selection, in contrast, was both pervasive and concordant: FUBAR identified purifying selection at 116 of 495 envelope codons, 41 of 352 in NS1, 128 of 618 in NS3 and 201 of 900 in NS5, and FEL, the more conservative method, identified 124 sites in total. GARD detected no recombination breakpoints in the coding alignment, with the model without a breakpoint strongly preferred (c-AIC 9815.9 versus 10,629.1 for the best single-breakpoint model).

Two primary results emerge from this analysis and are reported in full. First, BUSTED detected gene-wide evidence of episodic selection in NS3 (*p* = 0.0002) and, marginally, in NS5 (*p* = 0.045), although no site-level method identified a corresponding site in either gene. While the signal in NS5 is plausibly inflated by extreme site-to-site synonymous rate variation, NS3 exhibited the lowest variation among all four genes yet yielded the strongest BUSTED signal. Consequently, the origin of the NS3 signal remained unresolved, and it was not interpreted here as evidence of adaptive evolution; resolving it would require an extension of the method that explicitly accounts for synonymous rate variation. Second, when applied to the complete polyprotein with clade A branches designated as foreground, RELAX indicated a significant difference in the intensity of selection (K = 1.22, *p* = 0.012). Because the background ratio is far below one, a selection intensity parameter above one denotes intensified purifying selection rather than positive selection, and aBSREL found no branch of clade A under episodic diversifying selection, in either the complete polyprotein or NS3. Clade A was therefore more strongly constrained than the rest of the tree, showing no evidence of adaptive divergence.

### 3.6. Molecular Characterization of Clade A and Primer Compatibility

**Figure 4:**
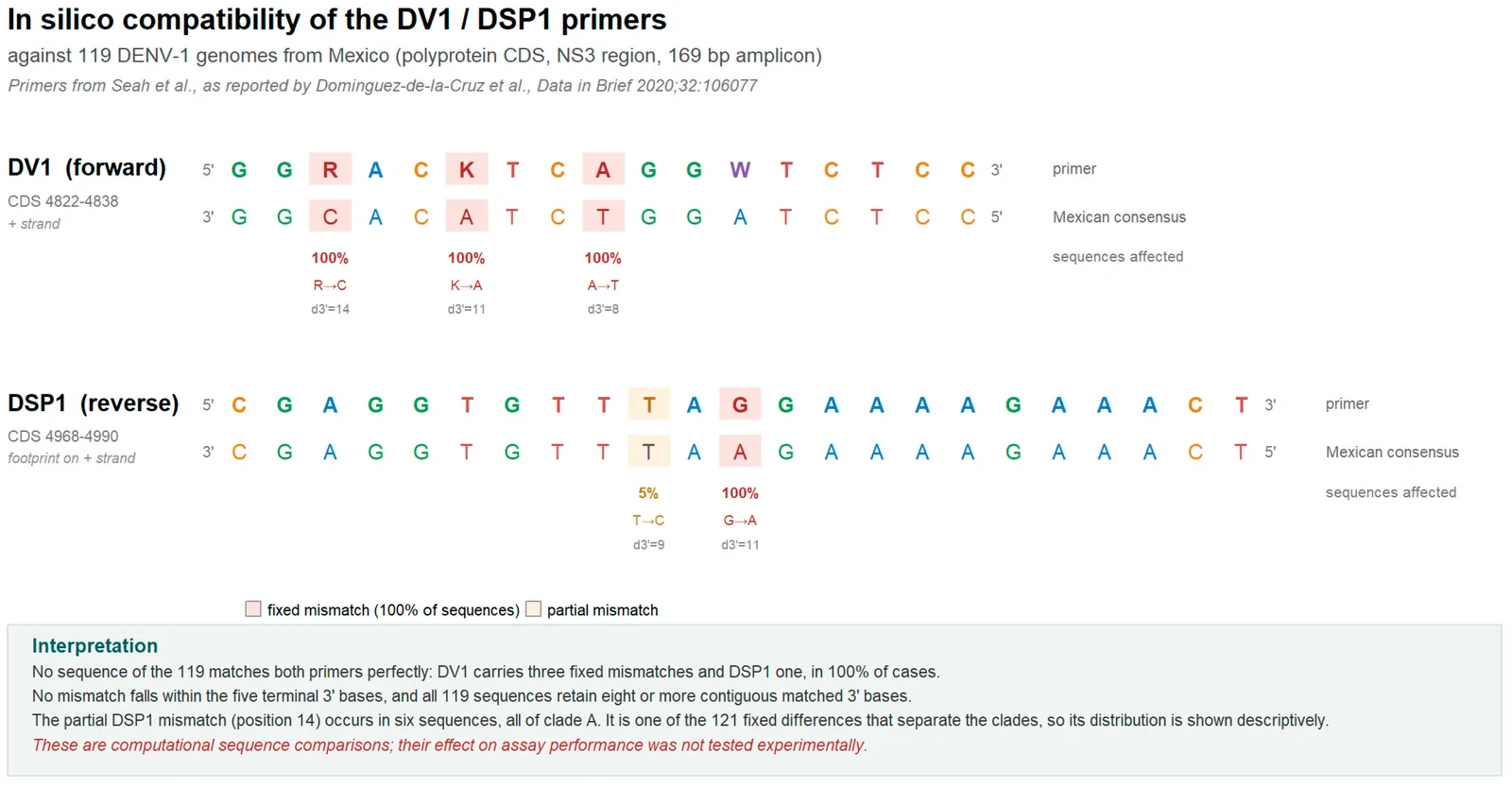
In silico compatibility of the DV1 and DSP1 serotyping primers with the 119 Mexican DENV-1 sequences. Primer and Mexican consensus sequences were aligned for each binding site; shaded columns indicate mismatches, along with the percentage of sequences affected and the distance to the primer 3′ terminus. The partial DSP1 mismatch was confined to the six clade A sequences [42,43].

Clade A was distinguished from clade B by 121 fixed nucleotide differences, of which 113 were synonymous and eight non-synonymous, a ratio of 14.1: 1 that was concordant with the genome-wide constraint reported above. The eight amino acid substitutions are listed in Table 4. The DV1 and DSP1 primer binding sites were located within NS3 at positions 4822 to 4838 and 4968 to 4990 of the coding sequence, yielding a predicted product of 169 bp identical to the published amplicon size (Figure 4). No sequence matched both primers perfectly: DV1 carried three mismatches in 100% of sequences, and DSP1 carried one, indicating that the degenerate positions incorporated in the primer design do not accommodate the Mexican genotype V variant. No mismatch occurred within the five 3′-terminal bases of either primer, and all 119 sequences exhibited at least eight contiguous matched bases at the 3′ end. These findings describe computational sequence compatibility; their effect on assay performance remains unvalidated, as no amplification experiments were performed. A second DSP1 mismatch was present in all six clade A sequences and absent from all 112 clade B sequences. This position is one of the 121 fixed differences distinguishing the two clades; its distribution is therefore presented descriptively and is not interpreted as independent confirmatory evidence of association. This mismatch is a synonymous transition at the third position of NS3 codon 184 and is therefore one of the substitutions distinguishing the clades: a change with no consequence at the protein level that nonetheless degrades complementarity with a widely used diagnostic primer.

### 3.7. Compatibility with Two Further Diagnostic Oligonucleotide Sets

The DENV-1 components of both additional assays were located in the alignment, and the products predicted from their primer positions reproduced the published sizes, providing a consistency check for the assignment of the binding sites. The three CDC oligonucleotides fell within NS5, at coding-sequence positions 8879–8898 (D1-F), 8967–8990 (D1-R) and 8904–8922 (D1-Probe), delimiting a 112 bp product with the probe internal to it. The C/prM oligonucleotides fell within the capsid and prM genes, at positions 38–65 (D1), 520–548 (D2) and 501–519 (TS1), giving a first-round product of 511 bp and a DENV-1 typing product of 482 bp. In every case, the runner-up window across both orientations carried at least 485 additional discordances over the panel.

Compatibility differed markedly between the three sets (Table 5). The consensus primer D1 of the C/prM assay was compatible with all 119 sequences at all 28 of its positions, the only oligonucleotide examined here with complete compatibility. Among the CDC oligonucleotides, 104 of 119 sequences (87.4%) were compatible with D1-F, 118 of 119 (99.2%) with D1-R, and 116 of 119 (97.5%) with the probe, of which 115 had fully resolved target bases; all three were simultaneously compatible in 100 sequences (84.0%), and each discordant sequence carried a single discordant position per oligonucleotide. The remaining oligonucleotides of the conventional and the C/prM sets were discordant with every sequence examined, but the number of discordant positions per sequence was smaller than the number of distinct positions observed across the panel: three per sequence for DV1, one or two for DSP1, four to six for D2, and one to three for TS1.

The position of the discordances matters more than their number. No CDC discordance fell within the five 3′-terminal bases of either primer, and 118 of 119 sequences retained at least eight contiguous compatible bases at the 3′ end of both. In the C/prM set, the pattern is different: the type-specific primer TS1 was discordant with every sequence, and the position of the discordance depended on lineage. In the 112 clade B sequences, it fell five bases from the 3′ terminus, at coding-sequence position 506, whereas in the six clade A sequences it fell at the 3′-terminal base itself, at position 501. Both positions are among the 121 fixed differences reported in Section 3.6, and position 506 corresponds to the substitution prM Q55R listed in Table 4. Among the CDC oligonucleotides, the most frequent discordance was that of D1-F, a synonymous transition at the third position of NS5 codon 471, carried by 15 sequences comprising all six of clade A and nine of the 112 of clade B. That position is not among the 121 fixed differences. Because sequences within a clade share ancestry and are not independent replicates, these distributions are presented descriptively and are not interpreted as independent confirmatory evidence of association. The single D1-R discordance and the two discordant probe positions, which affect three sequences in total, were synonymous changes.

These proportions describe sequence compatibility within a historical and unevenly sampled panel. They do not estimate diagnostic sensitivity, limit of detection, or the frequency of false-negative results, and these compatibility predictions were not tested experimentally.

## 4. Discussion

### 4.1. Genotype V Structure and Indications of Local Circulation

The Mexican DENV-1 sequences analyzed in this study all belong to genotype V and are structured into a single dominant clade with two temporal subclades, along with one independently introduced isolate. Nucleotide diversity in this Mexican panel is roughly an order of magnitude lower than values obtained from globally sampled multi-genotype panels [48,49], which highlights the importance of defining the population before reporting diversity indices. This configuration contrasts with the most closely comparable published dataset: Rojas-Gallardo et al. [5] analyzed DENV-1 genotype V in Risaralda and Valle del Cauca, Colombia, and resolved three clades, each most closely related to viruses from neighboring Colombian departments sampled over the preceding 5 to 10 years, and concluded that the 2019 outbreak reflected local dispersion rather than the introduction of a new lineage. Differences in geographic and temporal coverage limit direct comparison with the Colombian study. In the Mexican panel, the concentration of sampling in 2006–2009 and the absence of state-level annotations for 50 sequences limit the interpretation of geographic structure and sustained local circulation.

The two clades recovered here could be matched to an earlier Mexican report. Gonzalez-Duran et al. [3] described the co-circulation in Mexico of two lineages of genotype V between 2012 and 2014, one of which they reported for the first time in the country. Four partial NS5 sequences deposited with that study, all from InDRE isolates collected in 2013 and 2014, were assigned here using the 121 positions that distinguish the two clades. Two of them (KY347012 and KY347014, both from 2014) carried the clade A allele at every one of the fifteen diagnostic positions their fragment covered and the clade B allele at none, while the remaining two (KY347008 from 2013 and KY347010 from 2014) showed the complete opposite pattern. Clade A therefore corresponds to the lineage newly reported in that study, and clade B to the lineage with which it co-circulated. Two consequences follow. First, the sequences examined here represent clade A in 2011, 2013, 2014, 2015, 2016 and 2019. Gaps in this sampling record do not establish the absence of the lineage during the intervening years. Second, the inferred divergence predates the lineage’s first description by several decades. This does not establish when the lineage arrived in Mexico or whether it circulated continuously in the country during the intervening period.

Placing the Mexican sequences in regional context sharpens this comparison. The Colombian clades were each most closely related to viruses from neighboring Colombian departments, that is, to other sequences from the same country. The Mexican sequences show a different pattern: they are not monophyletic, and both Mexican clades have their closest sampled relatives outside Mexico, in Nicaragua and the United States. Cross-border introductions and subsequent local spread of DENV-1 have been described in other settings, including Guangdong, China [50]. Within our sampled dataset, the relationships among Mexican and regional sequences do not establish the immediate geographic source, direction of dispersal or continuity of circulation of either lineage. The diagnostic interpretation remains limited to the sequences directly evaluated; regional relatedness alone does not establish that the same compatibility patterns occur in other countries.

Three haplotypes shared between distinct Mexican isolates are consistent with local circulation, but do not by themselves establish sustained local transmission. A fourth candidate pair, involving an isolate designated Ciudad Juarez, could not be resolved because one of the two sequences carries an ambiguity at the single discriminating position; it is reported here as unresolved rather than as a shared haplotype. The Ciudad Juarez isolate is nonetheless informative for a different reason: it belongs to clade A, the lineage that separated from the dominant Mexican clade in the mid-1980s and that carries the DSP1 primer mismatch. Its record annotates the country of origin as Mexico without state-level detail, so the attribution to Chihuahua rests on the strain designation rather than on the structured metadata and should be treated accordingly.

The eight non-synonymous substitutions that distinguish clade A (Table 4) are distributed across six mature proteins, and their chemical character is consistent with the constraint inferred across the genome. Four are conservative exchanges between residues of similar character (E I55V, NS2A L91I and NS2B L4I between aliphatic residues, NS2B R91K between basic residues), and three replace polar residues with other polar or charged residues (prM Q17H, prM Q55R and NS1 N146D). Only NS5 S24P, a serine-to-proline substitution in the methyltransferase domain [51], replaces a residue with one of markedly different conformational properties. Two of the eight fall in regions of characterized function: NS2B R91K lies within the central hydrophilic region that acts as the cofactor of the NS3 protease [52], although it preserves the basic charge at that position, and E I55V lies at the interface between envelope domains I and II, outside the fusion loop [53] and away from the N-glycosylation sequons at E residues 67 and 153 [54].

Three of the eight substitutions fell in genes covered by the site-level analysis, and none showed concordant evidence of positive selection. E codon 55 and NS5 codon 24 returned no signal under any method (FEL *p* = 0.20 and 0.51; MEME *p* = 0.23 and 0.45; FUBAR posterior probabilities of diversifying selection 0.75 and 0.50). NS1 codon 146 reached the FUBAR threshold (posterior probability 0.90) but was supported by neither FEL (*p* = 0.39) nor MEME (*p* = 0.37) and was therefore one of the five sites flagged by a single method in Section 3.5. Against a genome-wide ω of 0.037 and a ratio of 113 synonymous to eight non-synonymous fixed differences, these substitutions are best interpreted as markers that record the divergence of clade A rather than as evidence of adaptive change. Establishing whether any of them alters protein function would require expression and functional assays beyond the scope of a sequence-based study.

### 4.2. Implications for Molecular Diagnostics

The finding with the most direct potential operational consequence concerns diagnostic primers, although it is entirely computational. The DV1/DSP1 pair carries fixed mismatches against every Mexican sequence examined, and a further mismatch is restricted to clade A. The findings for this primer pair describe computational sequence compatibility and are based solely on sequence complementarity. Their effect on assay performance remains unvalidated. No amplification-efficiency, limit-of-detection, or melting-temperature experiments were performed, and the DV1/DSP1 pair was not re-tested against clade A templates. The mismatch is therefore an unvalidated computational prediction and should not be read as evidence of reduced diagnostic sensitivity; experimental confirmation with clade A templates, ideally including a quantified limit-of-detection comparison against clade B, would be required before it could inform assay selection. Notably, the clade A mismatch is synonymous, which is worth emphasizing, and the codon-model analyses reinforce the point: no site in any of the four genes examined showed concordant evidence of diversifying selection. This is consistent with the strong purifying selection previously reported for dengue virus in Mexico [55]. Moreover, clade A itself exhibits intensified rather than relaxed purifying constraint. The sequence comparisons document the observed compatibility pattern, but they do not establish its evolutionary cause or its effect on assay performance. Whether this mismatch is present in currently circulating virus cannot be determined from these data: clade A is the only Mexican lineage represented in the panel after 2012, its most recent genome was collected in 2019, and no Mexican DENV-1 genome from a later collection year was retrieved by the query described in Section 2.1. The pattern observed in this historical panel does not establish its current frequency or its distribution outside the sequences evaluated. We suggest periodic in silico revalidation using tools such as PrimerProspector ver. 1.0.1. [56] or equivalent screening tools; the present study does not establish an optimal revalidation interval.

The three assays evaluated here place the finding in perspective. The comparison is between oligonucleotide sets with different targets and different design dates, so it does not by itself establish the benefit of any revalidation practice. What it does show is that discordance with the Mexican panel is a property of a particular oligonucleotide set rather than a general feature of these viruses. The CDC oligonucleotides, whose degenerate positions were selected against a global sequence panel, were simultaneously compatible with 84% of the sequences and carried no discordance fixed across the panel, whereas the conventional DV1/DSP1 pair and the C/prM primers D2 and TS1 were discordant with every sequence examined. The consensus primer D1 of the C/prM set was compatible with all 119, which shows that an oligonucleotide designed in 1992 can remain fully complementary to sequences collected up to 2019 when it targets a sufficiently conserved region. This analysis is consistent with the diagnostic surveillance objectives described in the 2025 WHO laboratory testing guidance [45].

Two observations bear on the interpretation. First, the 84% figure is the proportion of sequences compatible with all three oligonucleotides of one assay; it is not an estimate of diagnostic sensitivity, and it does not imply that the remaining infections would be missed. Second, the discordance with the clearest mechanistic implication is not the one reported in Section 3.6 but the 3′-terminal discordance of the type-specific primer TS1 in the six clade A sequences, because a mismatch at the 3′ terminus bears on primer extension in a way that an internal mismatch does not. All of these remain computational predictions. None was tested experimentally, and confirmation with clade A templates would be required before any of them could inform assay selection. The three sets examined were chosen as a conventional system, a published real-time reference assay, and the historical protocol cited in WHO guidance; they do not cover every assay in present use.

### 4.3. Implications for Genomic Surveillance

This study relied exclusively on publicly available data, which is central to its rationale. Sequences deposited by others were used to characterize lineage structure within the sampled Mexican panel, to date its divergence, to place it within regional circulation, and to detect a mismatch with a diagnostic primer. The placement of the isolate designated Ciudad Juarez illustrates the point: a virus sampled once could be assigned to a lineage whose inferred divergence predates its first description by decades, without generating a single new sequence. Where local sequencing capacity is limited, public repositories can therefore support genuine characterization rather than mere description, provided the sequences are curated with attention to reading frame, ambiguity content, and the definition of the population being analyzed.

These findings may inform priorities for the molecular surveillance of dengue in Mexico. The identification of a lineage-associated primer mismatch underscores the need for periodic in silico revalidation of diagnostic primers against currently circulating sequences, a practice that could be integrated into the routine activities of the national reference laboratory (InDRE). Furthermore, the gaps in genomic coverage identified here—particularly the uneven state-level coverage and the absence of state-level annotations for 50 sequences—highlight priority regions for future sampling within Mexico’s molecular surveillance program.

Three practical recommendations follow. First, whole-genome sequencing should be prioritized where feasible because it provides broader genomic context for interpreting lineage relationships and sequence variation, while partial sequences can still support lineage assignment. Genomic studies have also explored associations between viral variation and dengue disease severity [9]. Second, diagnostic primer and probe sets should be revalidated in silico against currently circulating sequences at regular intervals, because the substitution responsible for the mismatch described here is synonymous and therefore invisible to any analysis based on selection or protein change. The 2025 WHO laboratory testing guidance lists the identification of possible mutations affecting NAAT primers among the purposes of sequencing representative samples [45], although it does not prescribe a revalidation interval. Third, sequencing effort should be directed at the gaps this dataset exposes rather than at replicating its strengths: 91% of the available Mexican genomes come from a single outbreak period, one state dominates the sample, and no genomes are available from large parts of the country, including regions such as the Sierra Tarahumara, where the presence of *Aedes aegypti* has recently been documented [15].

These gaps are now addressable. Portable sequencing platforms and pan-serotype amplicon protocols such as DengueSeq [57] place whole-genome sequencing within reach of laboratories with modest infrastructure, and direct sequencing from NS1 rapid diagnostic tests [58] offers a route where cold-chain logistics are limiting. The analytical pipeline used here relies exclusively on open-source tools and is deposited with the Supplementary Material; is deposited with the Supplementary Material; genomic surveillance programs established under comparable constraints elsewhere illustrate what becomes feasible [59,60].

This study provides a documented historical reference for the sampled sequences, with explicit geographic and temporal limits. The uneven coverage and incomplete metadata constrain estimates of prevalence, geographic origin, and continuity of circulation. These limitations define the scope of the present conclusions and the information needed to interpret future observations alongside this dataset. The absence of sequences from remote or under-sampled regions (e.g., Indigenous communities and the Sierra Tarahumara) restricts our understanding of viral diversity in these populations. Future studies should include locally sequenced viruses to establish direct phylogenetic links [6]. Additionally, the use of consensus-level sequences may underestimate true viral diversity; haplotype-level sequencing (e.g., using Oxford Nanopore Technology) should be prioritized in future surveillance efforts [61].

A further limitation applies to the regional analysis. The context sequences were subsampled to prevent the best-sequenced countries from dominating, but the resulting sample remains uneven: 25 sequences were included from Nicaragua, which represents the per-country subsampling cap, and six sequences each from the United States and Colombia. The strongly supported clade uniting Mexican, Nicaraguan and United States sequences is therefore reliable, but the apparent affinity with Nicaragua may reflect sampling depth rather than epidemiological proximity and resolving the immediate geographic source of either Mexican clade would require targeted sequencing of the neighboring countries. In addition, 91% of the Mexican panel was collected between 2006 and 2009 and only 11 sequences date from 2011 onwards, so clade A rests on six genomes and its current prevalence cannot be estimated.

Two limitations apply to the selection analyses. The RELAX result rests on six clade A sequences whose laboratory provenance is heterogeneous: two were passaged once in C6/36 cells, one is annotated only with a mammalian cell line, one carries a passage-two designation, and one comes from a reference collection, whereas the comparison set is dominated by a single uniformly processed cohort passaged once or twice in C6/36. Differences in the intensity of selection between these groups cannot be clearly separated from differences in laboratory history, and the result should be read accordingly. In addition, the gene-wide signal detected by BUSTED in NS3 was not localized by any site-level method and its origin remains unresolved; distinguishing genuine diffuse episodic selection from inflation caused by synonymous rate variation would require a version of the test that models that variation explicitly.

## 5. Conclusions

DENV-1 sequences from Mexico showed a nucleotide diversity of 0.00831, an order of magnitude lower than values obtained from globally sampled multi-genotype panels, underscoring the importance of defining the population before reporting diversity indices. All 119 sequences belong to genotype V. Within the Mexican panel, they resolve into two well-supported clades plus one independently introduced isolate; placed among sequences from 19 other American countries and territories, however, they are not monophyletic, and both clades have their closest sampled relatives in Nicaragua and the United States, indicating participation in a regional circulation pool rather than an isolated national lineage. Three haplotypes shared between distinct Mexican isolates are consistent with local circulation. Molecular clock analysis dates the most recent common ancestor of Mexican DENV-1 to 1977, coinciding with the introduction of the serotype into the Americas, and shows that the two Mexican clades separated in the mid-1980s; clade A therefore corresponds to a lineage whose inferred divergence dates to the mid-1980s and whose earliest genome in the analyzed panel was collected in 2011. Selection analyses using three site-level methods across 2365 codons found no site with concordant evidence of diversifying selection, while purifying selection was pervasive, and clade A showed evidence of intensified rather than relaxed constraint; no recombination was detected. Finally, a synonymous mismatch in the DSP1 serotyping primer binding site is restricted to clade A and absent from the earlier clade, illustrating how a substitution with no consequence at the protein level, and arising under no detectable selection, can nonetheless reduce predicted primer complementarity; because that lineage extends beyond Mexico, the potential implication is regional. This mismatch was identified in silico and was not tested experimentally; its effect on assay performance, if any, remains to be established. Diagnostic primer sets should be periodically revalidated in silico against currently circulating sequences. These conclusions reflect the currently available Mexican sequences rather than the country as a whole: 91% of the dataset was collected between 2006 and 2009, 66 of 119 sequences originate from Yucatan, and clade A is represented by only six genomes, so its current prevalence cannot be reliably estimated. Therefore, geographically and temporally balanced sequencing is required to resolve the introduction history and dispersal patterns of DENV-1 in Mexico.

## Figures and Tables

**Table 1:** Genetic diversity indices of DENV-1 sequences.

Parameter	Value
Number of sequences (N)	119
Number of haplotypes (h)	115
Haplotype diversity (Hd)	0.9994
Nucleotide diversity (π)	0.00831
Polymorphic sites (S)	1225
Average nucleotide differences (k)	83.7

**Table 2:** Neutrality tests for DENV-1 sequences.

Test	Value
Tajima’s D	−2.18 (*p* = 0.009)
Fu and Li’s D*	−4.38 (*p* < 0.05)
Fu and Li’s F*	−4.03 (*p* < 0.05)
Fu’s Fs	−45.33 (see caveat in text)

Note. D* and F* denote the Fu and Li statistics computed without an outgroup; the asterisk is part of the statistic name and does not indicate statistical significance. These statistics were computed following the conventions of DnaSP v6.12.03 [30], which bases them on the total number of mutations; the implementation was validated by reproducing the published DnaSP output for the original panel. The significance of D* and F* was assessed against the critical values of Fu and Li [28]; both exceeded the two-tailed 5% threshold. Fu’s Fs is reported for completeness and is not interpreted as independent evidence of demographic expansion.

**Table 3:** Synonymous (dS) and non-synonymous (dN) substitution rates by mature protein.

Gene	Codons	dN	dS	dN/dS (ω)
**C**	114	0.00216	0.01610	0.134
**prM**	166	0.00211	0.03510	0.060
**E**	490	0.00080	0.03504	0.023
**NS1**	352	0.00069	0.04052	0.017
**NS2A**	216	0.00374	0.02953	0.127
**NS2B**	130	0.00257	0.05049	0.051
**NS3**	617	0.00082	0.03858	0.021
**NS4A**	127	0.00145	0.03988	0.036
**2K**	23	0.00033	0.01197	0.028
**NS4B**	249	0.00015	0.02795	0.005
**NS5**	867	0.00123	0.02989	0.041
**Complete polyprotein**	3351	0.00124	0.03386	0.037

The substitution rates by mature protein were estimated by the Nei-Gojobori method with Jukes-Cantor correction over all 7021 pairwise comparisons among the 119 Mexican sequences. Codons containing an ambiguous base in any sequence were excluded. Therefore, the codons report the number of codons analyzed for each mature protein rather than its total length: of the 3392 codons in the polyprotein coding sequence (10,179 nt including the stop codon), 41 contained an ambiguous base in at least one sequence and were excluded, leaving the 3351 codons analyzed here.

**Table 4:** Amino acid substitutions distinguishing clade A from clade B, with positions given relative to the mature protein and the polyprotein.

Protein	Substitution	Polyprotein Position
**prM**	Q17H	131
**prM**	Q55R	169
**E**	I55V	335
**NS1**	N146D	921
**NS2A**	L91I	1218
**NS2B**	L4I	1349
**NS2B**	R91K	1436
**NS5**	S24P	2516

**Table 5:** In silico compatibility of three DENV-1 diagnostic oligonucleotide sets with the 119 Mexican sequences.

Oligonucleotide	Role	Length (nt)	Position	Compatible, n (%)	Distinct Discordant Positions Across the Panel	Discordant Positions per Sequence	Contiguous 3′ Compatible Bases, Minimum
**NS3 conventional serotyping; 169 bp product.** [42].							
DV1	Forward primer	17	4822–4838	0 (0)	3	3	8
DSP1	Reverse primer	23	4968–4990	0 (0)	2	1–2	9
**DENV-1–4 Real-Time RT-PCR; 112 bp product.** [44].							
D1-F	Forward primer	20	8879–8898	104 (87.4)	1	0–1	9
D1-R	Reverse primer	24	8967–8990	118 (99.2)	1	0–1	6
D1-Probe	Hydrolysis probe	19	8904–8922	116 (97.5); 115 with resolved target	2	0–1	n.a.
**C/prM nested RT-PCR; 511 bp and 482 bp products.** [47].							
D1	Consensus forward primer	28	38–65	119 (100)	0	0	28
D2	Consensus reverse primer	29	520–548	0 (0)	7	4–6	5
TS1	DENV-1 type-specific primer	19	501–519	0 (0)	5	1–3	0

Positions are given in the coordinates of the polyprotein coding sequence. Compatibility was assessed using a set-based criterion: a position was counted as compatible when every possible target base was permitted by the oligonucleotide, so that a degenerate target matching a degenerate oligonucleotide is not scored as a discordance. The two count columns use different units: distinct discordant positions are those seen anywhere in the panel, whereas the number of discordant positions per sequence is reported as a range across individual sequences. For the hydrolysis probe, the count of contiguous 3′ bases are not reported, because that terminus carries the quencher and is not extended. n.a., not applicable.

## Data Availability

The genomic sequences analyzed in this study are publicly available in the GenBank database (NCBI); accession numbers are provided in Supplementary Table S1. The codon alignments, the maximum-likelihood trees, the complete R and HyPhy analysis code, the output of the selection analyses, and the Supplementary Tables are archived at Zenodo: https://doi.org/10.5281/zenodo.22650189.

## Supplementary Materials

The following supporting information can be downloaded at: https://www.mdpi.com/article/10.3390/tropicalmed11100287/s1, Table S1: Accession numbers, collection years and states of the 119 Mexican DENV-1 sequences analyzed; Table S2: Accession numbers of the 14 DENV-1 genotype reference strains and of the DENV-2 outgroup; Table S3: The 121 nucleotide positions distinguishing clade A from clade B, with codon context, amino acid consequence and the allele carried by MT899083.1; Table S4: Per-sequence mismatch counts against the DV1 and DSP1 serotyping primers, with the number of contiguous matched bases at the 3′ end; Table S5: Accession numbers, countries and collection years of the 111 American context sequences; Table S6: Site-level output of FEL, MEME and FUBAR for the envelope, NS1, NS3 and NS5 genes (2365 codons). Provided as a separate data file, Table_S6_site_level_selection.csv, because of its length; Table S7: Substitution rates and root ages obtained in the twenty date-randomization replicates, with the observed data shown as replicate 0; Table S8: Clade assignment of the partial NS5 sequences of Gonzalez-Duran et al. at the diagnostic positions; Figure S1: Root-to-tip regression of genetic distance against year of collection for the 119 Mexican sequences, showing the temporal signal that supports clock-based dating (slope 5.89 × 10^−4^ substitutions per site per year, R^2^ = 0.77). Clade B is shown in blue and clade A in red.

## Abbreviations

The following abbreviations are used in this manuscript:

2K	2K peptide, the signal sequence between NS4A and NS4B
aBSREL	adaptive branch-site random effects likelihood
BUSTED	branch-site unrestricted statistical test for episodic diversification
C	capsid protein
CDS	coding sequence
CI	confidence interval
DENV	dengue virus
DENV-1	dengue virus serotype 1
DENV-2	dengue virus serotype 2
dN	number of non-synonymous substitutions per non-synonymous site
DnaSP	DNA sequence polymorphism, software for population genetic analysis
dS	number of synonymous substitutions per synonymous site
DSP1	reverse primer of the DV1/DSP1 serotyping pair
DV1	forward primer of the DV1/DSP1 serotyping pair
E	envelope protein
FEL	fixed-effects likelihood
FUBAR	fast unconstrained Bayesian approximation
GARD	genetic algorithm for recombination detection
Hd	haplotype diversity
HyPhy	hypothesis testing using phylogenies, software for codon-model analysis
IQ-TREE	software for maximum-likelihood phylogenetic inference
LSD2	least-squares dating, version 2
MEME	mixed-effects model of evolution
MUSCLE	multiple sequence comparison by log-expectation
NCBI	National Center for Biotechnology Information
NS1 to NS5	non-structural proteins 1 to 5
prM	precursor membrane protein
RELAX	test for relaxed intensity of natural selection
RT-PCR	reverse transcription polymerase chain reaction
SH-aLRT	Shimodaira–Hasegawa-like approximate likelihood ratio test
UFBoot	ultrafast bootstrap approximation
π	nucleotide diversity
ω	ratio of dN to dS
