# Supplementary material for: Genomic Diversity, Phylogenetic Structure, and In Silico Diagnostic Primer Compatibility of Available DENV-1 Genotype V from Mexico
Source: Trop Med Infect Dis. 2026 Oct 12;11(10):287. doi: 10.3390/tropicalmed11100287 (PMC13709455; doi:10.3390/tropicalmed11100287)
Supplement: Supplementary file 1 [file tropicalmed-11-00287-s001.zip › tropicalmed-4583962-supplementary.pdf]

# Supplementary Materials

## Genomic Diversity, Phylogenetic Structure, and In Silico Diagnostic Primer Compatibility of Available DENV-1 Genotype V from Mexico

Francisco Joel Cabrera-Santos, Fátima Yedith Camacho Sánchez, Sorayda Leonela González Trujillo, Miguel Angel Reyes López and Erick de Jesús De Luna Santillana 1,

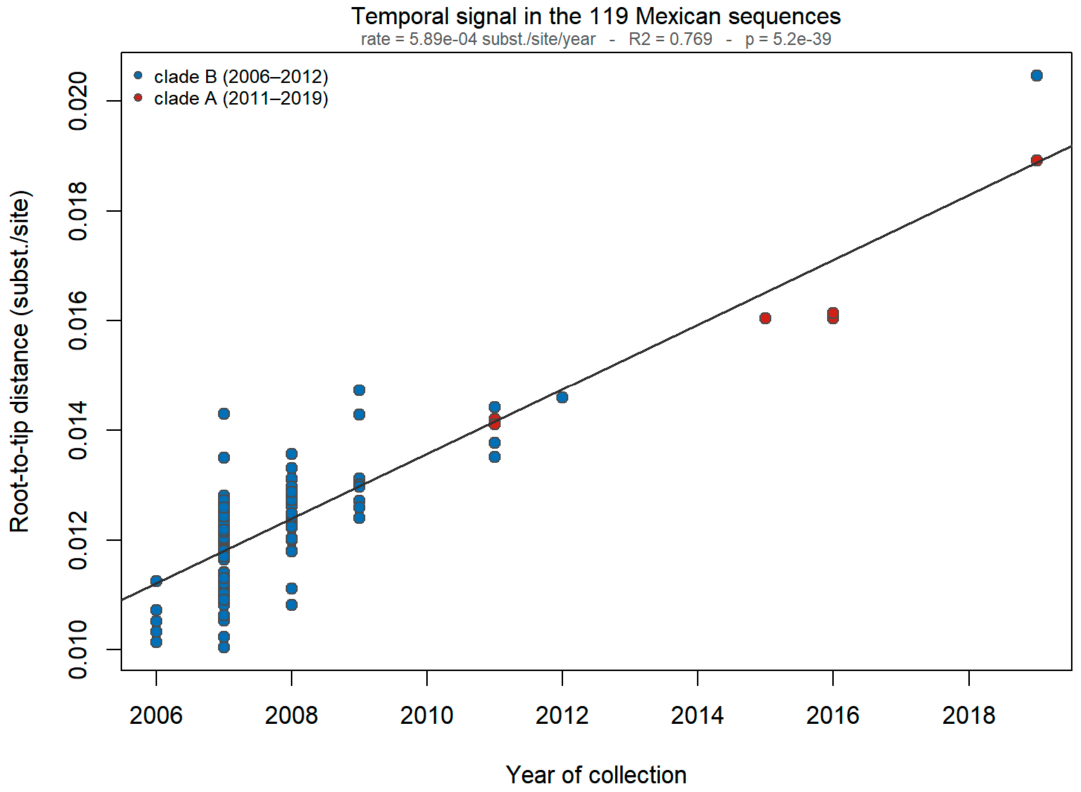


**Figure S1.** Root-to-tip regression of genetic distance against year of collection for the 119 Mexican sequences, showing the temporal signal that supports clock-based dating (slope 5.89 × $10^{-4}$ substitutions per site per year, $R^2$ = 0.77). Clade B is shown in blue and clade A in red.

**Table S1.** Accession numbers, collection years and states of the 119 Mexican DENV-1 sequences analyzed.

| Accession | Collection year | State | Ambiguous positions | Clade |
|---|---|---|---|---|
| **KJ189348.1** | 2011 | not specified | 0 | A |
| **KJ189349.1** | 2011 | not specified | 0 | A |
| **MZ343259.1** | 2015 | not specified | 0 | A |
| **OR389010.1** | 2016 | not specified | 12 | A |
| **PX282464.1** | 2016 | not specified | 1 | A |
| **MT929160.1** | 2019 | Jalisco | 0 | A |
| **GQ868498.1** | 2006 | Yucatan | 0 | B |
| **GQ868499.1** | 2006 | Quintana Roo | 0 | B |
| **GU131956.1** | 2006 | Yucatan | 0 | B |
| **GU131957.1** | 2006 | Yucatan | 0 | B |
| **GU131958.1** | 2006 | Yucatan | 0 | B |
| **GQ868500.1** | 2007 | Yucatan | 0 | B |

| Accession | Collection year | State | Ambiguous positions | Clade |
|---|---|---|---|---|
| **GQ868501.1** | 2007 | Yucatan | 0 | B |
| **GQ868502.1** | 2007 | Yucatan | 0 | B |
| **GQ868503.1** | 2007 | Yucatan | 0 | B |
| **GQ868504.1** | 2007 | Yucatan | 0 | B |
| **GQ868505.1** | 2007 | Yucatan | 0 | B |
| **GQ868506.1** | 2007 | Yucatan | 0 | B |
| **GQ868507.1** | 2007 | Yucatan | 0 | B |
| **GQ868508.1** | 2007 | Yucatan | 0 | B |
| **GQ868509.1** | 2007 | Yucatan | 0 | B |
| **GQ868510.1** | 2007 | Yucatan | 0 | B |
| **GQ868511.1** | 2007 | Yucatan | 0 | B |
| **GQ868512.1** | 2007 | Yucatan | 0 | B |
| **GQ868513.1** | 2007 | Yucatan | 0 | B |
| **GQ868514.1** | 2007 | Yucatan | 0 | B |
| **GQ868517.1** | 2007 | Yucatan | 0 | B |
| **GQ868518.1** | 2007 | Yucatan | 0 | B |
| **GQ868519.1** | 2007 | Yucatan | 0 | B |
| **GQ868520.1** | 2007 | Yucatan | 0 | B |
| **GQ868521.1** | 2007 | Yucatan | 0 | B |
| **GQ868522.1** | 2007 | Yucatan | 0 | B |
| **GQ868523.1** | 2007 | Yucatan | 0 | B |
| **GQ868524.1** | 2007 | Yucatan | 0 | B |
| **GQ868525.1** | 2007 | Yucatan | 0 | B |
| **GQ868526.1** | 2007 | Yucatan | 0 | B |
| **GQ868527.1** | 2007 | Yucatan | 0 | B |
| **GQ868528.1** | 2007 | Yucatan | 0 | B |
| **GU131960.1** | 2007 | Yucatan | 0 | B |
| **GU131961.1** | 2007 | Yucatan | 0 | B |
| **GU131963.1** | 2007 | Yucatan | 0 | B |
| **GU131964.1** | 2007 | Yucatan | 0 | B |
| **GU131965.1** | 2007 | Yucatan | 0 | B |
| **GU131966.1** | 2007 | Yucatan | 0 | B |
| **GU131967.1** | 2007 | Yucatan | 0 | B |
| **GU131968.1** | 2007 | Yucatan | 0 | B |
| **GU131969.1** | 2007 | Yucatan | 0 | B |
| **GU131970.1** | 2007 | Yucatan | 0 | B |
| **GU131971.1** | 2007 | Yucatan | 0 | B |
| **GU131972.1** | 2007 | Yucatan | 0 | B |
| **GU131973.1** | 2007 | Yucatan | 0 | B |
| **GU131976.1** | 2007 | Yucatan | 0 | B |
| **GU131977.1** | 2007 | Yucatan | 0 | B |
| **GU131978.1** | 2007 | Yucatan | 0 | B |
| **GU131979.1** | 2007 | Yucatan | 0 | B |
| **GU131980.1** | 2007 | Yucatan | 0 | B |
| **GU131981.1** | 2007 | Yucatan | 0 | B |
| **HQ166035.1** | 2007 | Yucatan | 0 | B |
| **HQ166036.1** | 2007 | Yucatan | 0 | B |
| **KF955427.1** | 2007 | not specified | 0 | B |
| **KF955442.1** | 2007 | not specified | 0 | B |
| **KF955443.1** | 2007 | not specified | 0 | B |
| **KJ189317.1** | 2007 | not specified | 0 | B |
| **KJ189318.1** | 2007 | not specified | 0 | B |
| **KJ189319.1** | 2007 | not specified | 0 | B |
| **KJ189320.1** | 2007 | not specified | 0 | B |
| **KJ189321.1** | 2007 | not specified | 0 | B |
| **KJ189322.1** | 2007 | not specified | 0 | B |

| Accession | Collection year | State | Ambiguous positions | Clade |
|---|---|---|---|---|
| **KJ189323.1** | 2007 | not specified | 0 | B |
| **KJ189324.1** | 2007 | not specified | 0 | B |
| **KJ189325.1** | 2007 | not specified | 0 | B |
| **KJ189326.1** | 2007 | not specified | 0 | B |
| **KJ189327.1** | 2007 | not specified | 0 | B |
| **KJ189328.1** | 2007 | not specified | 0 | B |
| **KJ189329.1** | 2007 | not specified | 0 | B |
| **KJ189330.1** | 2007 | not specified | 0 | B |
| **GQ868529.1** | 2008 | Yucatan | 0 | B |
| **GQ868530.1** | 2008 | Yucatan | 0 | B |
| **GQ868531.1** | 2008 | Yucatan | 0 | B |
| **GQ868532.1** | 2008 | Yucatan | 0 | B |
| **GQ868533.1** | 2008 | Yucatan | 0 | B |
| **GQ868534.1** | 2008 | Yucatan | 0 | B |
| **GQ868535.1** | 2008 | Yucatan | 0 | B |
| **GQ868536.1** | 2008 | Yucatan | 0 | B |
| **GQ868537.1** | 2008 | Yucatan | 0 | B |
| **GQ868538.1** | 2008 | Yucatan | 0 | B |
| **GQ868539.1** | 2008 | Yucatan | 0 | B |
| **GU131982.1** | 2008 | not specified | 0 | B |
| **GU131983.1** | 2008 | Yucatan | 0 | B |
| **GU131984.1** | 2008 | Yucatan | 0 | B |
| **HQ166037.1** | 2008 | Yucatan | 0 | B |
| **KF955433.1** | 2008 | not specified | 83 | B |
| **KJ189312.1** | 2008 | not specified | 0 | B |
| **KJ189313.1** | 2008 | not specified | 0 | B |
| **KJ189314.1** | 2008 | not specified | 0 | B |
| **KJ189315.1** | 2008 | not specified | 0 | B |
| **KJ189331.1** | 2008 | not specified | 0 | B |
| **KJ189332.1** | 2008 | not specified | 0 | B |
| **KJ189333.1** | 2008 | not specified | 0 | B |
| **KJ189334.1** | 2008 | not specified | 0 | B |
| **KJ189335.1** | 2008 | not specified | 0 | B |
| **KJ189336.1** | 2008 | not specified | 0 | B |
| **KJ189337.1** | 2008 | not specified | 0 | B |
| **KJ189338.1** | 2008 | not specified | 0 | B |
| **KJ189339.1** | 2008 | not specified | 0 | B |
| **KJ189340.1** | 2008 | not specified | 0 | B |
| **KJ189316.1** | 2009 | not specified | 0 | B |
| **KJ189341.1** | 2009 | not specified | 0 | B |
| **KJ189342.1** | 2009 | not specified | 0 | B |
| **KJ189343.1** | 2009 | not specified | 0 | B |
| **KJ189344.1** | 2009 | not specified | 0 | B |
| **KJ189345.1** | 2009 | not specified | 0 | B |
| **KJ189346.1** | 2009 | not specified | 0 | B |
| **KJ189347.1** | 2009 | not specified | 0 | B |
| **KJ189306.1** | 2011 | not specified | 0 | B |
| **KJ189307.1** | 2011 | not specified | 0 | B |
| **KJ189369.1** | 2011 | not specified | 0 | B |
| **KJ189368.1** | 2012 | not specified | 0 | B |
| **MT899083.1** | 2019 | Jalisco | 0 | unassigned |

**Table S2.** Accession numbers of the 14 DENV-1 genotype reference strains and of the DENV-2 outgroup.

| accession | genotype | role |
|---|---|---|
| **KY672938.1** | GTI | genotype reference |
| **KY672937.1** | GTI | genotype reference |
| **KY672936.1** | GTI | genotype reference |
| **JN544411.2** | GTII | genotype reference |
| **JN544410.2** | GTII | genotype reference |
| **JN544409.2** | GTIII | genotype reference |
| **KY921903.1** | GTIII | genotype reference |
| **JN544407.2** | GTIII | genotype reference |
| **KY057371.1** | GTIV | genotype reference |
| **KY057368.1** | GTIV | genotype reference |
| **KY057366.1** | GTIV | genotype reference |
| **PQ140589.1** | GTV | genotype reference |
| **PQ140591.1** | GTV | genotype reference |
| **PQ140590.1** | GTV | genotype reference |
| **NC_001474.2** | DENV-2 | outgroup |

**Table S3.** The 121 nucleotide positions distinguishing clade A from clade B, with codon context, amino acid consequence and the allele carried by MT899083.1.

| cds_position | gene | polyprotein_residue | pos_codon | nt_B | nt_A | codon_B | codon_A | aa_B | aa_A | type | substitution_polyprotein | MT899083 | residue_in_protein | substitution_in_protein |
|---|---|---|---|---|---|---|---|---|---|---|---|---|---|---|
| **109** | C | 37 | 1 | T | C | TTG | CTG | L | L | synonymous | | T | 37 | |
| **315** | C | 105 | 3 | T | C | CTT | CTC | L | L | synonymous | | C | 105 | |
| **360** | prM | 120 | 3 | A | G | CGA | CGG | R | R | synonymous | | A | 6 | |
| **393** | prM | 131 | 3 | G | T | CAG | CAT | Q | H | non-synonymous | Q131H | G | 17 | prM Q17H |
| **438** | prM | 146 | 3 | T | C | AAT | AAC | N | N | synonymous | | T | 32 | |
| **501** | prM | 167 | 3 | C | T | TGC | TGT | C | C | synonymous | | C | 53 | |
| **506** | prM | 169 | 2 | A | G | CAG | CGG | Q | R | non-synonymous | Q169R | G | 55 | prM Q55R |
| **633** | prM | 211 | 3 | A | G | CCA | CCG | P | P | synonymous | | A | 97 | |
| **759** | prM | 253 | 3 | T | C | TTT | TTC | F | F | synonymous | | T | 139 | |
| **816** | prM | 272 | 3 | G | A | CTG | CTA | L | L | synonymous | | G | 158 | |
| **936** | E | 312 | 3 | T | C | ACT | ACC | T | T | synonymous | | C | 32 | |
| **1003** | E | 335 | 1 | A | G | ATC | GTC | I | V | non-synonymous | I335V | G | 55 | E I55V |
| **1155** | E | 385 | 3 | T | C | TGT | TGC | C | C | synonymous | | C | 105 | |
| **1245** | E | 415 | 3 | A | G | TTA | TTG | L | L | synonymous | | A | 135 | |
| **1368** | E | 456 | 3 | C | T | ACC | ACT | T | T | synonymous | | C | 176 | |
| **1419** | E | 473 | 3 | T | C | TTT | TTC | F | F | synonymous | | C | 193 | |
| **1686** | E | 562 | 3 | T | C | CAT | CAC | H | H | synonymous | | C | 282 | |
| **1687** | E | 563 | 1 | C | T | CTG | TTG | L | L | synonymous | | C | 283 | |
| **1788** | E | 596 | 3 | G | A | CAG | CAA | Q | Q | synonymous | | G | 316 | |
| **1887** | E | 629 | 3 | G | A | GGG | GGA | G | G | synonymous | | G | 349 | |

| cds_position | gene | polyprotein_residue | pos_codon | nt_B | nt_A | codon_B | codon_A | aa_B | aa_A | type | substitution_polyprotein | MT899083 | residue_in_protein | substitution_in_protein |
|---|---|---|---|---|---|---|---|---|---|---|---|---|---|---|
| **1914** | E | 638 | 3 | C | T | GTC | GTT | V | V | synonymous | | T | 358 | |
| **2001** | E | 667 | 3 | A | G | TTA | TTG | L | L | synonymous | | G | 387 | |
| **2055** | E | 685 | 3 | C | T | ACC | ACT | T | T | synonymous | | C | 405 | |
| **2082** | E | 694 | 3 | T | C | ATT | ATC | I | I | synonymous | | C | 414 | |
| **2106** | E | 702 | 3 | T | C | TTT | TTC | F | F | synonymous | | C | 422 | |
| **2112** | E | 704 | 3 | C | T | TCC | TCT | S | S | synonymous | | T | 424 | |
| **2136** | E | 712 | 3 | G | A | GTG | GTA | V | V | synonymous | | G | 432 | |
| **2202** | E | 734 | 3 | T | C | ACT | ACC | T | T | synonymous | | C | 454 | |
| **2268** | E | 756 | 3 | G | A | TCG | TCA | S | S | synonymous | | G | 476 | |
| **2301** | E | 767 | 3 | A | G | CTA | CTG | L | L | synonymous | | G | 487 | |
| **2364** | NS1 | 788 | 3 | C | T | CTC | CTT | L | L | synonymous | | T | 13 | |
| **2446** | NS1 | 816 | 1 | C | A | CGA | AGA | R | R | synonymous | | A | 41 | |
| **2761** | NS1 | 921 | 1 | A | G | AAC | GAC | N | D | non-synonymous | N921D | G | 146 | NS1 N146D |
| **2830** | NS1 | 944 | 1 | T | C | TTG | CTG | L | L | synonymous | | C | 169 | |
| **2959** | NS1 | 987 | 1 | C | T | CTG | TTG | L | L | synonymous | | C | 212 | |
| **3180** | NS1 | 1060 | 3 | C | T | GTC | GTT | V | V | synonymous | | T | 285 | |
| **3213** | NS1 | 1071 | 3 | T | A | CCT | CCA | P | P | synonymous | | A | 296 | |
| **3231** | NS1 | 1077 | 3 | A | G | ACA | ACG | T | T | synonymous | | A | 302 | |
| **3369** | NS1 | 1123 | 3 | A | G | TCA | TCG | S | S | synonymous | | A | 348 | |
| **3411** | NS2A | 1137 | 3 | A | G | TTA | TTG | L | L | synonymous | | A | 10 | |
| **3652** | NS2A | 1218 | 1 | C | A | CTA | ATA | L | I | non-synonymous | L1218I | C | 91 | NS2A L91I |
| **3657** | NS2A | 1219 | 3 | A | G | TTA | TTG | L | L | synonymous | | G | 92 | |

| cds_position | gene | polyprotein_residue | pos_codon | nt_B | nt_A | codon_B | codon_A | aa_B | aa_A | type | substitution_polyprotein | MT899083 | residue_in_protein | substitution_in_protein |
|---|---|---|---|---|---|---|---|---|---|---|---|---|---|---|
| **3709** | NS2A | 1237 | 1 | T | C | TTG | CTG | L | L | synonymous | | C | 110 | |
| **3723** | NS2A | 1241 | 3 | G | A | GTG | GTA | V | V | synonymous | | G | 114 | |
| **3843** | NS2A | 1281 | 3 | C | T | TTC | TTT | F | F | synonymous | | T | 154 | |
| **3990** | NS2A | 1330 | 3 | G | A | CCG | CCA | P | P | synonymous | | A | 203 | |
| **3996** | NS2A | 1332 | 3 | T | C | ACT | ACC | T | T | synonymous | | C | 205 | |
| **4045** | NS2B | 1349 | 1 | C | A | CTC | ATC | L | I | non-synonymous | L1349I | C | 4 | NS2B L4I |
| **4077** | NS2B | 1359 | 3 | T | C | GTT | GTC | V | V | synonymous | | T | 14 | |
| **4089** | NS2B | 1363 | 3 | A | G | CTA | CTG | L | L | synonymous | | A | 18 | |
| **4227** | NS2B | 1409 | 3 | G | A | GAG | GAA | E | E | synonymous | | A | 64 | |
| **4239** | NS2B | 1413 | 3 | C | A | TCC | TCA | S | S | synonymous | | C | 68 | |
| **4258** | NS2B | 1420 | 1 | C | T | CTA | TTA | L | L | synonymous | | T | 75 | |
| **4307** | NS2B | 1436 | 2 | G | A | AGG | AAG | R | K | non-synonymous | R1436K | G | 91 | NS2B R91K |
| **4401** | NS2B | 1467 | 3 | C | T | TAC | TAT | Y | Y | synonymous | | T | 122 | |
| **4404** | NS2B | 1468 | 3 | T | C | TTT | TTC | F | F | synonymous | | T | 123 | |
| **4524** | NS3 | 1508 | 3 | G | A | AGG | AGA | R | R | synonymous | | G | 33 | |
| **4533** | NS3 | 1511 | 3 | A | G | GTA | GTG | V | V | synonymous | | A | 36 | |
| **4650** | NS3 | 1550 | 3 | T | C | GAT | GAC | D | D | synonymous | | C | 75 | |
| **4788** | NS3 | 1596 | 3 | T | C | GGT | GGC | G | G | synonymous | | C | 121 | |
| **4794** | NS3 | 1598 | 3 | T | C | GTT | GTC | V | V | synonymous | | T | 123 | |
| **4923** | NS3 | 1641 | 3 | C | T | GCC | GCT | A | A | synonymous | | C | 166 | |
| **4977** | NS3 | 1659 | 3 | T | C | TTT | TTC | F | F | synonymous | | T | 184 | |
| **5043** | NS3 | 1681 | 3 | A | G | CCA | CCG | P | P | synonymous | | A | 206 | |

| cds_position | gene | polyprotein_residue | pos_codon | nt_B | nt_A | codon_B | codon_A | aa_B | aa_A | type | substitution_polyprotein | MT899083 | residue_in_protein | substitution_in_protein |
|---|---|---|---|---|---|---|---|---|---|---|---|---|---|---|
| **5073** | NS3 | 1691 | 3 | A | G | AAA | AAG | K | K | synonymous | | A | 216 | |
| **5199** | NS3 | 1733 | 3 | T | A | GTT | GTA | V | V | synonymous | | T | 258 | |
| **5412** | NS3 | 1804 | 3 | C | T | AGC | AGT | S | S | synonymous | | C | 329 | |
| **5520** | NS3 | 1840 | 3 | T | C | AGT | AGC | S | S | synonymous | | C | 365 | |
| **5544** | NS3 | 1848 | 3 | T | C | GCT | GCC | A | A | synonymous | | C | 373 | |
| **5631** | NS3 | 1877 | 3 | C | T | GAC | GAT | D | D | synonymous | | T | 402 | |
| **5865** | NS3 | 1955 | 3 | A | G | TTA | TTG | L | L | synonymous | | A | 480 | |
| **5871** | NS3 | 1957 | 3 | T | C | AAT | AAC | N | N | synonymous | | T | 482 | |
| **6066** | NS3 | 2022 | 3 | G | A | CTG | CTA | L | L | synonymous | | A | 547 | |
| **6072** | NS3 | 2024 | 3 | T | C | TAT | TAC | Y | Y | synonymous | | C | 549 | |
| **6241** | NS3 | 2081 | 1 | T | C | TTG | CTG | L | L | synonymous | | C | 606 | |
| **6330** | NS4A | 2110 | 3 | T | C | CAT | CAC | H | H | synonymous | | T | 17 | |
| **6339** | NS4A | 2113 | 3 | A | G | TTA | CTG | L | L | synonymous | | G | 20 | |
| **6414** | NS4A | 2138 | 3 | C | T | GCC | GCT | A | A | synonymous | | T | 45 | |
| **6531** | NS4A | 2177 | 3 | C | T | ATC | ATT | I | I | synonymous | | T | 84 | |
| **6535** | NS4A | 2179 | 1 | C | T | CTG | TTG | L | L | synonymous | | T | 86 | |
| **6567** | NS4A | 2189 | 3 | G | A | TTG | TTA | L | L | synonymous | | A | 96 | |
| **6624** | NS4A | 2208 | 3 | T | C | TTT | TTC | F | F | synonymous | | T | 115 | |
| **6639** | NS4A | 2213 | 3 | T | C | CTT | CTC | L | L | synonymous | | C | 120 | |
| **6786** | NS4B | 2262 | 3 | A | G | GTA | GTG | V | V | synonymous | | A | 19 | |
| **6810** | NS4B | 2270 | 3 | T | C | GCT | GCC | A | A | synonymous | | T | 27 | |
| **7008** | NS4B | 2336 | 3 | T | C | GTT | GTC | V | V | synonymous | | T | 93 | |
| **7065** | NS4B | 2355 | 3 | G | A | GCG | GCA | A | A | synonymous | | G | 112 | |

| cds_position | gene | polyprotein_residue | pos_codon | nt_B | nt_A | codon_B | codon_A | aa_B | aa_A | type | substitution_polyprotein | MT899083 | residue_in_protein | substitution_in_protein |
|---|---|---|---|---|---|---|---|---|---|---|---|---|---|---|
| **7191** | NS4B | 2397 | 3 | A | T | GCA | GCT | A | A | synonymous | | A | 154 | |
| **7252** | NS4B | 2418 | 1 | T | C | TTA | CTA | L | L | synonymous | | T | 175 | |
| **7275** | NS4B | 2425 | 3 | G | A | CAG | CAA | Q | Q | synonymous | | G | 182 | |
| **7278** | NS4B | 2426 | 3 | C | T | ATC | ATT | I | I | synonymous | | C | 183 | |
| **7326** | NS4B | 2442 | 3 | T | G | GCT | GCG | A | A | synonymous | | T | 199 | |
| **7452** | NS4B | 2484 | 3 | A | G | TTA | TTG | L | L | synonymous | | A | 241 | |
| **7546** | NS5 | 2516 | 1 | T | C | TCA | CCA | S | P | non-synonymous | S2516P | T | 24 | NS5 S24P |
| **7575** | NS5 | 2525 | 3 | A | G | GGA | GGG | G | G | synonymous | | G | 33 | |
| **7614** | NS5 | 2538 | 3 | G | A | CTG | CTA | L | L | synonymous | | G | 46 | |
| **7731** | NS5 | 2577 | 3 | A | G | AGA | AGG | R | R | synonymous | | A | 85 | |
| **7909** | NS5 | 2637 | 1 | C | T | CTG | TTG | L | L | synonymous | | C | 145 | |
| **7995** | NS5 | 2665 | 3 | A | C | CTA | CTC | L | L | synonymous | | C | 173 | |
| **8157** | NS5 | 2719 | 3 | C | T | AAC | AAT | N | N | synonymous | | C | 227 | |
| **8355** | NS5 | 2785 | 3 | C | T | CAC | CAT | H | H | synonymous | | T | 293 | |
| **8439** | NS5 | 2813 | 3 | G | A | GTG | GTA | V | V | synonymous | | G | 321 | |
| **8619** | NS5 | 2873 | 3 | A | G | TTA | TTG | L | L | synonymous | | A | 381 | |
| **8778** | NS5 | 2926 | 3 | C | T | CTC | CTT | L | L | synonymous | | C | 434 | |
| **8784** | NS5 | 2928 | 3 | C | T | CAC | CAT | H | H | synonymous | | C | 436 | |
| **9054** | NS5 | 3018 | 3 | G | A | CCG | CCA | P | P | synonymous | | A | 526 | |
| **9087** | NS5 | 3029 | 3 | T | A | GGT | GGA | G | G | synonymous | | A | 537 | |
| **9174** | NS5 | 3058 | 3 | G | A | ACG | ACA | T | T | synonymous | | G | 566 | |
| **9192** | NS5 | 3064 | 3 | T | C | ACT | ACC | T | T | synonymous | | T | 572 | |

| cds_position | gene | polyprotein_residue | pos_codon | nt_B | nt_A | codon_B | codon_A | aa_B | aa_A | type | substitution_polyprotein | MT899083 | residue_in_protein | substitution_in_protein |
|---|---|---|---|---|---|---|---|---|---|---|---|---|---|---|
| **9201** | NS5 | 3067 | 3 | C | T | AAC | AAT | N | N | synonymous | | C | 575 | |
| **9204** | NS5 | 3068 | 3 | A | G | AAA | AAG | K | K | synonymous | | G | 576 | |
| **9234** | NS5 | 3078 | 3 | C | T | AAC | AAT | N | N | synonymous | | T | 586 | |
| **9258** | NS5 | 3086 | 3 | C | T | TCC | TCT | S | S | synonymous | | C | 594 | |
| **9501** | NS5 | 3167 | 3 | T | A | GCT | GCA | A | A | synonymous | | A | 675 | |
| **9516** | NS5 | 3172 | 3 | C | T | GCC | GCT | A | A | synonymous | | T | 680 | |
| **9675** | NS5 | 3225 | 3 | A | G | GAA | GAG | E | E | synonymous | | A | 733 | |
| **9681** | NS5 | 3227 | 3 | G | A | GTG | GTA | V | V | synonymous | | G | 735 | |
| **9766** | NS5 | 3256 | 1 | C | T | CTG | TTG | L | L | synonymous | | C | 764 | |
| **9867** | NS5 | 3289 | 3 | C | T | ATC | ATT | I | I | synonymous | | C | 797 | |
| **10059** | NS5 | 3353 | 3 | T | C | ACT | ACC | T | T | synonymous | | C | 861 | |
| **10178** | stop | 3393 | 2 | G | A | TGA | TAA | * | * | synonymous | | A | NA | |

**Table S4.** Per-sequence mismatch counts against the DV1 and DSP1 serotyping primers, with the number of contiguous matched bases at the 3′ end.

| Accession | State | Collection year | Mismatches DV1 | Contiguous 3prime DV1 | Mismatches DSP1 | Contiguous 3prime DSP1 | Mismatches total | Clade |
|---|---|---|---|---|---|---|---|---|
| **KJ189348.1** | not specified | 2011 | 3 | 8 | 2 | 9 | 5 | A |
| **KJ189349.1** | not specified | 2011 | 3 | 8 | 2 | 9 | 5 | A |
| **MT929160.1** | Jalisco | 2019 | 3 | 8 | 2 | 9 | 5 | A |
| **MZ343259.1** | not specified | 2015 | 3 | 8 | 2 | 9 | 5 | A |
| **OR389010.1** | not specified | 2016 | 3 | 8 | 2 | 9 | 5 | A |
| **PX282464.1** | not specified | 2016 | 3 | 8 | 2 | 9 | 5 | A |
| **GQ868498.1** | Yucatan | 2006 | 3 | 8 | 1 | 11 | 4 | B |
| **GQ868499.1** | Quintana Roo | 2006 | 3 | 8 | 1 | 11 | 4 | B |
| **GQ868500.1** | Yucatan | 2007 | 3 | 8 | 1 | 11 | 4 | B |
| **GQ868501.1** | Yucatan | 2007 | 3 | 8 | 1 | 11 | 4 | B |
| **GQ868502.1** | Yucatan | 2007 | 3 | 8 | 1 | 11 | 4 | B |
| **GQ868503.1** | Yucatan | 2007 | 3 | 8 | 1 | 11 | 4 | B |
| **GQ868504.1** | Yucatan | 2007 | 3 | 8 | 1 | 11 | 4 | B |
| **GQ868505.1** | Yucatan | 2007 | 3 | 8 | 1 | 11 | 4 | B |
| **GQ868506.1** | Yucatan | 2007 | 3 | 8 | 1 | 11 | 4 | B |
| **GQ868507.1** | Yucatan | 2007 | 3 | 8 | 1 | 11 | 4 | B |
| **GQ868508.1** | Yucatan | 2007 | 3 | 8 | 1 | 11 | 4 | B |
| **GQ868509.1** | Yucatan | 2007 | 3 | 8 | 1 | 11 | 4 | B |
| **GQ868510.1** | Yucatan | 2007 | 3 | 8 | 1 | 11 | 4 | B |
| **GQ868511.1** | Yucatan | 2007 | 3 | 8 | 1 | 11 | 4 | B |
| **GQ868512.1** | Yucatan | 2007 | 3 | 8 | 1 | 11 | 4 | B |
| **GQ868513.1** | Yucatan | 2007 | 3 | 8 | 1 | 11 | 4 | B |
| **GQ868514.1** | Yucatan | 2007 | 3 | 8 | 1 | 11 | 4 | B |

| Accession | State | Collection year | Mismatches DV1 | Contiguous 3prime DV1 | Mismatches DSP1 | Contiguous 3prime DSP1 | Mismatches total | Clade |
|---|---|---|---|---|---|---|---|---|
| **GQ868517.1** | Yucatan | 2007 | 3 | 8 | 1 | 11 | 4 | B |
| **GQ868518.1** | Yucatan | 2007 | 3 | 8 | 1 | 11 | 4 | B |
| **GQ868519.1** | Yucatan | 2007 | 3 | 8 | 1 | 11 | 4 | B |
| **GQ868520.1** | Yucatan | 2007 | 3 | 8 | 1 | 11 | 4 | B |
| **GQ868521.1** | Yucatan | 2007 | 3 | 8 | 1 | 11 | 4 | B |
| **GQ868522.1** | Yucatan | 2007 | 3 | 8 | 1 | 11 | 4 | B |
| **GQ868523.1** | Yucatan | 2007 | 3 | 8 | 1 | 11 | 4 | B |
| **GQ868524.1** | Yucatan | 2007 | 3 | 8 | 1 | 11 | 4 | B |
| **GQ868525.1** | Yucatan | 2007 | 3 | 8 | 1 | 11 | 4 | B |
| **GQ868526.1** | Yucatan | 2007 | 3 | 8 | 1 | 11 | 4 | B |
| **GQ868527.1** | Yucatan | 2007 | 3 | 8 | 1 | 11 | 4 | B |
| **GQ868528.1** | Yucatan | 2007 | 3 | 8 | 1 | 11 | 4 | B |
| **GQ868529.1** | Yucatan | 2008 | 3 | 8 | 1 | 11 | 4 | B |
| **GQ868530.1** | Yucatan | 2008 | 3 | 8 | 1 | 11 | 4 | B |
| **GQ868531.1** | Yucatan | 2008 | 3 | 8 | 1 | 11 | 4 | B |
| **GQ868532.1** | Yucatan | 2008 | 3 | 8 | 1 | 11 | 4 | B |
| **GQ868533.1** | Yucatan | 2008 | 3 | 8 | 1 | 11 | 4 | B |
| **GQ868534.1** | Yucatan | 2008 | 3 | 8 | 1 | 11 | 4 | B |
| **GQ868535.1** | Yucatan | 2008 | 3 | 8 | 1 | 11 | 4 | B |
| **GQ868536.1** | Yucatan | 2008 | 3 | 8 | 1 | 11 | 4 | B |
| **GQ868537.1** | Yucatan | 2008 | 3 | 8 | 1 | 11 | 4 | B |
| **GQ868538.1** | Yucatan | 2008 | 3 | 8 | 1 | 11 | 4 | B |
| **GQ868539.1** | Yucatan | 2008 | 3 | 8 | 1 | 11 | 4 | B |
| **GU131956.1** | Yucatan | 2006 | 3 | 8 | 1 | 11 | 4 | B |
| **GU131957.1** | Yucatan | 2006 | 3 | 8 | 1 | 11 | 4 | B |
| **GU131958.1** | Yucatan | 2006 | 3 | 8 | 1 | 11 | 4 | B |
| **GU131960.1** | Yucatan | 2007 | 3 | 8 | 1 | 11 | 4 | B |

| Accession | State | Collection year | Mismatches DV1 | Contiguous 3prime DV1 | Mismatches DSP1 | Contiguous 3prime DSP1 | Mismatches total | Clade |
|---|---|---|---|---|---|---|---|---|
| **GU131961.1** | Yucatan | 2007 | 3 | 8 | 1 | 11 | 4 | B |
| **GU131963.1** | Yucatan | 2007 | 3 | 8 | 1 | 11 | 4 | B |
| **GU131964.1** | Yucatan | 2007 | 3 | 8 | 1 | 11 | 4 | B |
| **GU131965.1** | Yucatan | 2007 | 3 | 8 | 1 | 11 | 4 | B |
| **GU131966.1** | Yucatan | 2007 | 3 | 8 | 1 | 11 | 4 | B |
| **GU131967.1** | Yucatan | 2007 | 3 | 8 | 1 | 11 | 4 | B |
| **GU131968.1** | Yucatan | 2007 | 3 | 8 | 1 | 11 | 4 | B |
| **GU131969.1** | Yucatan | 2007 | 3 | 8 | 1 | 11 | 4 | B |
| **GU131970.1** | Yucatan | 2007 | 3 | 8 | 1 | 11 | 4 | B |
| **GU131971.1** | Yucatan | 2007 | 3 | 8 | 1 | 11 | 4 | B |
| **GU131972.1** | Yucatan | 2007 | 3 | 8 | 1 | 11 | 4 | B |
| **GU131973.1** | Yucatan | 2007 | 3 | 8 | 1 | 11 | 4 | B |
| **GU131976.1** | Yucatan | 2007 | 3 | 8 | 1 | 11 | 4 | B |
| **GU131977.1** | Yucatan | 2007 | 3 | 8 | 1 | 11 | 4 | B |
| **GU131978.1** | Yucatan | 2007 | 3 | 8 | 1 | 11 | 4 | B |
| **GU131979.1** | Yucatan | 2007 | 3 | 8 | 1 | 11 | 4 | B |
| **GU131980.1** | Yucatan | 2007 | 3 | 8 | 1 | 11 | 4 | B |
| **GU131981.1** | Yucatan | 2007 | 3 | 8 | 1 | 11 | 4 | B |
| **GU131982.1** | not specified | 2008 | 3 | 8 | 1 | 11 | 4 | B |
| **GU131983.1** | Yucatan | 2008 | 3 | 8 | 1 | 11 | 4 | B |
| **GU131984.1** | Yucatan | 2008 | 3 | 8 | 1 | 11 | 4 | B |
| **HQ166035.1** | Yucatan | 2007 | 3 | 8 | 1 | 11 | 4 | B |
| **HQ166036.1** | Yucatan | 2007 | 3 | 8 | 1 | 11 | 4 | B |
| **HQ166037.1** | Yucatan | 2008 | 3 | 8 | 1 | 11 | 4 | B |
| **KF955427.1** | not specified | 2007 | 3 | 8 | 1 | 11 | 4 | B |
| **KF955433.1** | not specified | 2008 | 3 | 8 | 1 | 11 | 4 | B |

| Accession | State | Collection year | Mismatches DV1 | Contiguous 3prime DV1 | Mismatches DSP1 | Contiguous 3prime DSP1 | Mismatches total | Clade |
|---|---|---|---|---|---|---|---|---|
| **KF955442.1** | not specified | 2007 | 3 | 8 | 1 | 11 | 4 | B |
| **KF955443.1** | not specified | 2007 | 3 | 8 | 1 | 11 | 4 | B |
| **KJ189306.1** | not specified | 2011 | 3 | 8 | 1 | 11 | 4 | B |
| **KJ189307.1** | not specified | 2011 | 3 | 8 | 1 | 11 | 4 | B |
| **KJ189312.1** | not specified | 2008 | 3 | 8 | 1 | 11 | 4 | B |
| **KJ189313.1** | not specified | 2008 | 3 | 8 | 1 | 11 | 4 | B |
| **KJ189314.1** | not specified | 2008 | 3 | 8 | 1 | 11 | 4 | B |
| **KJ189315.1** | not specified | 2008 | 3 | 8 | 1 | 11 | 4 | B |
| **KJ189316.1** | not specified | 2009 | 3 | 8 | 1 | 11 | 4 | B |
| **KJ189317.1** | not specified | 2007 | 3 | 8 | 1 | 11 | 4 | B |
| **KJ189318.1** | not specified | 2007 | 3 | 8 | 1 | 11 | 4 | B |
| **KJ189319.1** | not specified | 2007 | 3 | 8 | 1 | 11 | 4 | B |
| **KJ189320.1** | not specified | 2007 | 3 | 8 | 1 | 11 | 4 | B |
| **KJ189321.1** | not specified | 2007 | 3 | 8 | 1 | 11 | 4 | B |
| **KJ189322.1** | not specified | 2007 | 3 | 8 | 1 | 11 | 4 | B |
| **KJ189323.1** | not specified | 2007 | 3 | 8 | 1 | 11 | 4 | B |
| **KJ189324.1** | not specified | 2007 | 3 | 8 | 1 | 11 | 4 | B |
| **KJ189325.1** | not specified | 2007 | 3 | 8 | 1 | 11 | 4 | B |

| Accession | State | Collection year | Mismatches DV1 | Contiguous 3prime DV1 | Mismatches DSP1 | Contiguous 3prime DSP1 | Mismatches total | Clade |
|---|---|---|---|---|---|---|---|---|
| **KJ189326.1** | not specified | 2007 | 3 | 8 | 1 | 11 | 4 | B |
| **KJ189327.1** | not specified | 2007 | 3 | 8 | 1 | 11 | 4 | B |
| **KJ189328.1** | not specified | 2007 | 3 | 8 | 1 | 11 | 4 | B |
| **KJ189329.1** | not specified | 2007 | 3 | 8 | 1 | 11 | 4 | B |
| **KJ189330.1** | not specified | 2007 | 3 | 8 | 1 | 11 | 4 | B |
| **KJ189331.1** | not specified | 2008 | 3 | 8 | 1 | 11 | 4 | B |
| **KJ189332.1** | not specified | 2008 | 3 | 8 | 1 | 11 | 4 | B |
| **KJ189333.1** | not specified | 2008 | 3 | 8 | 1 | 11 | 4 | B |
| **KJ189334.1** | not specified | 2008 | 3 | 8 | 1 | 11 | 4 | B |
| **KJ189335.1** | not specified | 2008 | 3 | 8 | 1 | 11 | 4 | B |
| **KJ189336.1** | not specified | 2008 | 3 | 8 | 1 | 11 | 4 | B |
| **KJ189337.1** | not specified | 2008 | 3 | 8 | 1 | 11 | 4 | B |
| **KJ189338.1** | not specified | 2008 | 3 | 8 | 1 | 11 | 4 | B |
| **KJ189339.1** | not specified | 2008 | 3 | 8 | 1 | 11 | 4 | B |
| **KJ189340.1** | not specified | 2008 | 3 | 8 | 1 | 11 | 4 | B |
| **KJ189341.1** | not specified | 2009 | 3 | 8 | 1 | 11 | 4 | B |
| **KJ189342.1** | not specified | 2009 | 3 | 8 | 1 | 11 | 4 | B |
| **KJ189343.1** | not specified | 2009 | 3 | 8 | 1 | 11 | 4 | B |

| Accession | State | Collection year | Mismatches DV1 | Contiguous 3prime DV1 | Mismatches DSP1 | Contiguous 3prime DSP1 | Mismatches total | Clade |
|---|---|---|---|---|---|---|---|---|
| **KJ189344.1** | not specified | 2009 | 3 | 8 | 1 | 11 | 4 | B |
| **KJ189345.1** | not specified | 2009 | 3 | 8 | 1 | 11 | 4 | B |
| **KJ189346.1** | not specified | 2009 | 3 | 8 | 1 | 11 | 4 | B |
| **KJ189347.1** | not specified | 2009 | 3 | 8 | 1 | 11 | 4 | B |
| **KJ189368.1** | not specified | 2012 | 3 | 8 | 1 | 11 | 4 | B |
| **KJ189369.1** | not specified | 2011 | 3 | 8 | 1 | 11 | 4 | B |
| **MT899083.1** | Jalisco | 2019 | 3 | 8 | 1 | 11 | 4 | unassigned |

**Table S5.** Accession numbers, countries and collection years of the 111 American context sequences.

| Accession | Country | Collection year |
|---|---|---|
| **KC692510.1** | Argentina | 2009 |
| **KC692509.1** | Argentina | 2009 |
| **KC692508.1** | Argentina | 2009 |
| **KC692517.1** | Argentina | 2010 |
| **KC692516.1** | Argentina | 2010 |
| **KC692515.1** | Argentina | 2010 |
| **MW946053.1** | Bolivia | 2010 |
| **PV206068.1** | Brazil | 2022 |
| **PV206067.1** | Brazil | 2022 |
| **PV206066.1** | Brazil | 2022 |
| **PV206072.1** | Brazil | 2023 |
| **PV206071.1** | Brazil | 2023 |
| **PV206070.1** | Brazil | 2023 |
| **PV206033.1** | Brazil | 2024 |
| **PV206032.1** | Brazil | 2024 |
| **PV206031.1** | Brazil | 2024 |
| **OR389275.1** | Colombia | 1988 |
| **OR389276.1** | Colombia | 1990 |
| **PX282462.1** | Colombia | 2004 |
| **OR389009.1** | Colombia | 2004 |
| **PV088275.1** | Colombia | 2011 |
| **OQ603268.1** | Colombia | 2016 |
| **OQ821332.1** | Costa Rica | NA |
| **OQ821351.1** | Cuba | NA |
| **OQ821349.1** | Cuba | NA |
| **PP234945.1** | Dominican Republic | NA |
| **OQ821358.1** | Dominican Republic | NA |
| **PX297361.1** | Ecuador | 1988 |
| **OR389277.1** | Ecuador | 1988 |
| **OK605756.1** | Ecuador | 2007 |
| **KY474307.1** | Ecuador | 2014 |

| Accession | Country | Collection year |
|---|---|---|
| **KY474306.1** | Ecuador | 2014 |
| **KY474305.1** | Ecuador | 2014 |
| **MN556095.1** | Ecuador | 2017 |
| **JN819417.1** | El Salvador | 1993 |
| **PP213459.1** | French Guiana | 2009 |
| **EF122232.1** | French Guiana | NA |
| **EF122231.1** | French Guiana | NA |
| **OR389280.1** | Guatemala | 1989 |
| **KU509264.1** | Haiti | 2010 |
| **MG877553.1** | Haiti | 2012 |
| **KT279761.2** | Haiti | 2014 |
| **OR389292.1** | Jamaica | 1977 |
| **GQ199875.1** | Nicaragua | 2004 |
| **GQ199873.1** | Nicaragua | 2004 |
| **GQ199872.1** | Nicaragua | 2004 |
| **JN819402.1** | Nicaragua | 2005 |
| **FJ873814.1** | Nicaragua | 2005 |
| **FJ850114.1** | Nicaragua | 2005 |
| **JN819403.1** | Nicaragua | 2006 |
| **FJ810419.1** | Nicaragua | 2006 |
| **FJ547068.1** | Nicaragua | 2006 |
| **FJ898433.1** | Nicaragua | 2007 |
| **GQ199859.1** | Nicaragua | 2008 |
| **GQ199858.1** | Nicaragua | 2008 |
| **GQ199857.1** | Nicaragua | 2008 |
| **JF937644.1** | Nicaragua | 2009 |
| **JF937645.1** | Nicaragua | 2009 |
| **JF937635.1** | Nicaragua | 2009 |
| **KF973462.1** | Nicaragua | 2011 |
| **KF973460.1** | Nicaragua | 2011 |
| **KF973459.1** | Nicaragua | 2011 |
| **KF973475.1** | Nicaragua | 2012 |
| **KF973474.1** | Nicaragua | 2012 |
| **KF973473.1** | Nicaragua | 2012 |
| **MZ008453.1** | Nicaragua | 2013 |
| **MZ008451.1** | Nicaragua | 2013 |
| **MZ008450.1** | Nicaragua | 2013 |
| **PZ655094.1** | Peru | NA |
| **PX490371.1** | Peru | NA |
| **PX490370.1** | Peru | NA |
| **KF921911.1** | Puerto Rico | 1996 |
| **PX282466.1** | Puerto Rico | 2006 |
| **OR389014.1** | Puerto Rico | 2006 |
| **MW945426.1** | Puerto Rico | 2006 |
| **KJ189367.1** | Puerto Rico | 2010 |
| **KJ189366.1** | Puerto Rico | 2010 |
| **KJ189365.1** | Puerto Rico | 2010 |
| **KJ189359.1** | Puerto Rico | 2012 |
| **KJ189358.1** | Puerto Rico | 2012 |
| **KJ189357.1** | Puerto Rico | 2012 |
| **OR389304.1** | Trinidad and Tobago | 1978 |
| **OR389303.1** | Trinidad and Tobago | 1978 |
| **MN244560.1** | Trinidad and Tobago | 2011 |
| **MN244559.1** | Trinidad and Tobago | 2011 |
| **MN244558.1** | Trinidad and Tobago | 2011 |
| **MN244563.1** | Trinidad and Tobago | 2012 |

| Accession | Country | Collection year |
|---|---|---|
| **MN244562.1** | Trinidad and Tobago | 2012 |
| **MN244561.1** | Trinidad and Tobago | 2012 |
| **MN244566.1** | Trinidad and Tobago | 2013 |
| **MN244565.1** | Trinidad and Tobago | 2013 |
| **MN244564.1** | Trinidad and Tobago | 2013 |
| **MN244556.1** | Trinidad and Tobago | 2014 |
| **MN244555.1** | Trinidad and Tobago | 2014 |
| **PX297368.1** | USA | 1944 |
| **PX726960.1** | USA | NA |
| **PX395888.1** | USA | NA |
| **PV833223.1** | USA | NA |
| **OR771173.1** | USA | NA |
| **OR771159.1** | USA | NA |
| **MW946058.1** | Venezuela | 2000 |
| **JN819425.1** | Venezuela | 2004 |
| **GU131836.1** | Venezuela | 2004 |
| **GU131835.1** | Venezuela | 2004 |
| **JN819412.1** | Venezuela | 2005 |
| **JN819411.1** | Venezuela | 2005 |
| **JN819415.1** | Venezuela | 2006 |
| **JN819413.1** | Venezuela | 2006 |
| **JN819414.1** | Venezuela | 2007 |
| **GU131842.1** | Venezuela | 2007 |
| **MH450312.1** | Venezuela | 2015 |

**Table S6.** Site-level output of FEL, MEME and FUBAR for the envelope, NS1, NS3 and NS5 genes (2,365 codons). Provided as a separate data file, Table_S6_site_level_selection.csv, because of its length.

**Table S7.** Substitution rates and root ages obtained in the twenty date-randomization replicates, with the observed data shown as replicate 0.

| Replicate | Substitution rate | Root age | Rate at optimizer lower bound |
|---|---|---|---|
| **0** | 0.0004611 | 1977.5 | no (observed data) |
| **1** | 1e-10 | -170758000 | yes |
| **2** | 7.644e-06 | -226.2 | no |
| **3** | 2.254e-05 | 1250.4 | no |
| **4** | 1e-10 | -170765000 | yes |
| **5** | 1e-10 | -170760000 | yes |
| **6** | 1.627e-05 | 960.1 | no |
| **7** | 5.231e-06 | -1257.5 | no |
| **8** | 1e-10 | -170760000 | yes |
| **9** | 1e-10 | -170773000 | yes |
| **10** | 1e-10 | -170763000 | yes |
| **11** | 1.257e-05 | 648.2 | no |
| **12** | 1e-10 | -170758000 | yes |
| **13** | 1e-10 | -170769000 | yes |
| **14** | 1e-10 | -170781000 | yes |
| **15** | 2.732e-05 | 1382.8 | no |
| **16** | 1e-10 | -170768000 | yes |
| **17** | 1e-10 | -170770000 | yes |
| **18** | 1e-10 | -170757000 | yes |
| **19** | 1e-10 | -170758000 | yes |
| **20** | 1e-10 | -170766000 | yes |

**Table S8.** Clade assignment of the partial NS5 sequences of Gonzalez-Duran et al. at the diagnostic positions.

| Accession | Strain | Collection year | Diagnostic_positions_with_clade_A_allele | Diagnostic_positions_with_clade_B_allele | Positions_not_covered_by_the_fragment | Assignment |
|---|---|---|---|---|---|---|
| **KY347014.1** | DENV-1/MX/InDRE_36C/2014 | 2014 | 15 | 0 | 105 | clade A |
| **KY347012.1** | DENV-1/MX/InDRE_35B/2014 | 2014 | 15 | 0 | 105 | clade A |
| **KY347010.1** | DENV-1/MX/InDRE_33B/2014 | 2014 | 0 | 17 | 103 | clade B |
| **KY347008.1** | DENV-1/MX/InDRE_27B/2013 | 2013 | 0 | 13 | 107 | clade B |
